# Solid‐State Photochemistry in Resonant Acoustic Mixers

**DOI:** 10.1002/chem.202501137

**Published:** 2025-06-12

**Authors:** Carolina Spula, Phil M. Preuß, Lars Borchardt, Sven Grätz

**Affiliations:** ^1^ Inorganic Chemistry I, Ruhr‐Universität Bochum Universitätsstr. 150 44801 Bochum Germany

**Keywords:** mechanochemistry, photochemistry, photoreactor, resonant acoustic mixer, sustainable chemistry

## Abstract

Herein, we developed the first photochemical reaction set‐up under solid‐state conditions using a resonant acoustic mixer (RAM), a novel mechanochemical methodology that eliminates the need for milling media. This innovation outscores previous approaches in the ball mill under avoidance of milling balls and their related disadvantages. Further, it enables the facile performance and upscaling of photochemical reactions up to a factor of 10. We investigate the influences of liquid and solid additives on the rheology and performance of two model reactions and demonstrate and compare the efficiency and applicability of two custom‐designed photoreactors.

## Graphical abstract

Bringing light to solid‐state photochemistry – We introduce the first photochemical reaction using a resonant acoustic mixer (RAM), eliminating the need for milling media. This innovative, solvent‐free approach enables scalable photochemistry under UVC light. Therefore, we designed and compared two custom‐made photoreactors, paving the way for greener, more efficient photochemical reactions.

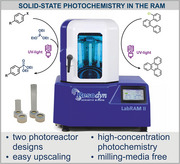

## 1 Introduction

Light is one of the most environment‐friendly activation modes, being both renewable and waste‐free. However, conventional photochemical reactions suffer from sustainability issues, as they are often performed in highly diluted solutions due to the poor penetration depth of photons which results in substantial waste production.^[^ ^1^, ^2^ ^]^ Owing to the global incentives toward environmentally friendly synthesis alternatives, research on solvent‐free pathways is increasing.^[^ ^3^, ^4^ ^]^ Nonetheless, typical photochemical approaches in the solid‐state are performed on glass plates suffering from poor diffusion and requiring small sample batches. Mechanochemistry is an alternative solid‐state synthesis strategy capable of overcoming these disadvantages. Mechanochemical synthesis describes the performance of chemical reactions under the impact of mechanical energy avoiding bulk solvents,^[^ ^5^, ^6^ ^]^ providing greener and more efficient reactions,^[^ ^4a^ ^,–^ ^f^ ^]^ and also opening the pathways toward otherwise inaccessible products.^[^ ^13^, ^14^ ^]^ Next to the limited amount of publications on combining photochemistry and ball milling,^[^ ^6^, ^7^ ^]^ our working group has recently addressed the challenge by designing translucent quartz glass milling vials, opening up the opportunities for photochemical reactions with higher energy UVC light.^[^ ^8^, ^9^, ^10^ ^]^ This innovation has overcome a major hurdle that enables efficient photochemical organic transformations without the use of metal catalysts and bulk solvents.^[^ ^7^, ^11^ ^]^ While these milling vessels are suitable for use with soft milling media made from polymers, quartz glass is not resistant to harder materials. In mechanochemistry, the usage of ball mills often results in abrasion caused by high energetic impacts leading to contamination of the product.^[^ ^12^ ^]^ For this reason, the research on milling media‐free technologies increased rapidly in the mechanochemical community. Here, the mechanical energy is implemented by the acceleration of the powder itself, using either ball mills in absence of milling media or devices such as Resonant Acoustic Mixer (RAM).^[^ ^13^, ^14^, ^15^, ^16^, ^17^, ^18^, ^19^ ^]^ This latter technique has recently emerged as an innovative methodology employing high‐frequency acoustic vibrations to induce intensified mixing of reactants, leading to improved reaction rates and reduced reaction times. Additionally, the avoidance of milling media reduces contamination of the reaction mixture. The energy input can be adjusted in terms of the magnitudes of the *g*‐forces, allowing for optimization of the reaction conditions. Next to various cocrystallization reactions,^[^ ^20a–c^ ^]^ recent publications demonstrate the efficient performance of mechanochemical synthesis within the RAM including metal‐catalyzed transformations,^[^ ^13^, ^15^, ^16^ ^]^ biosynthesis,^[^ ^17^ ^]^ mechanoredox catalysis,^[^ ^18^ ^]^ and metal‐organic framework (MOF) synthesis.^[^ ^19^ ^]^ To ensure sufficient molecular‐level mixing in the absence of milling balls the use of liquid assisted grinding (LAG) facilitates the product formation.^[^ ^13^, ^14^ ^]^

**Figure 1:**
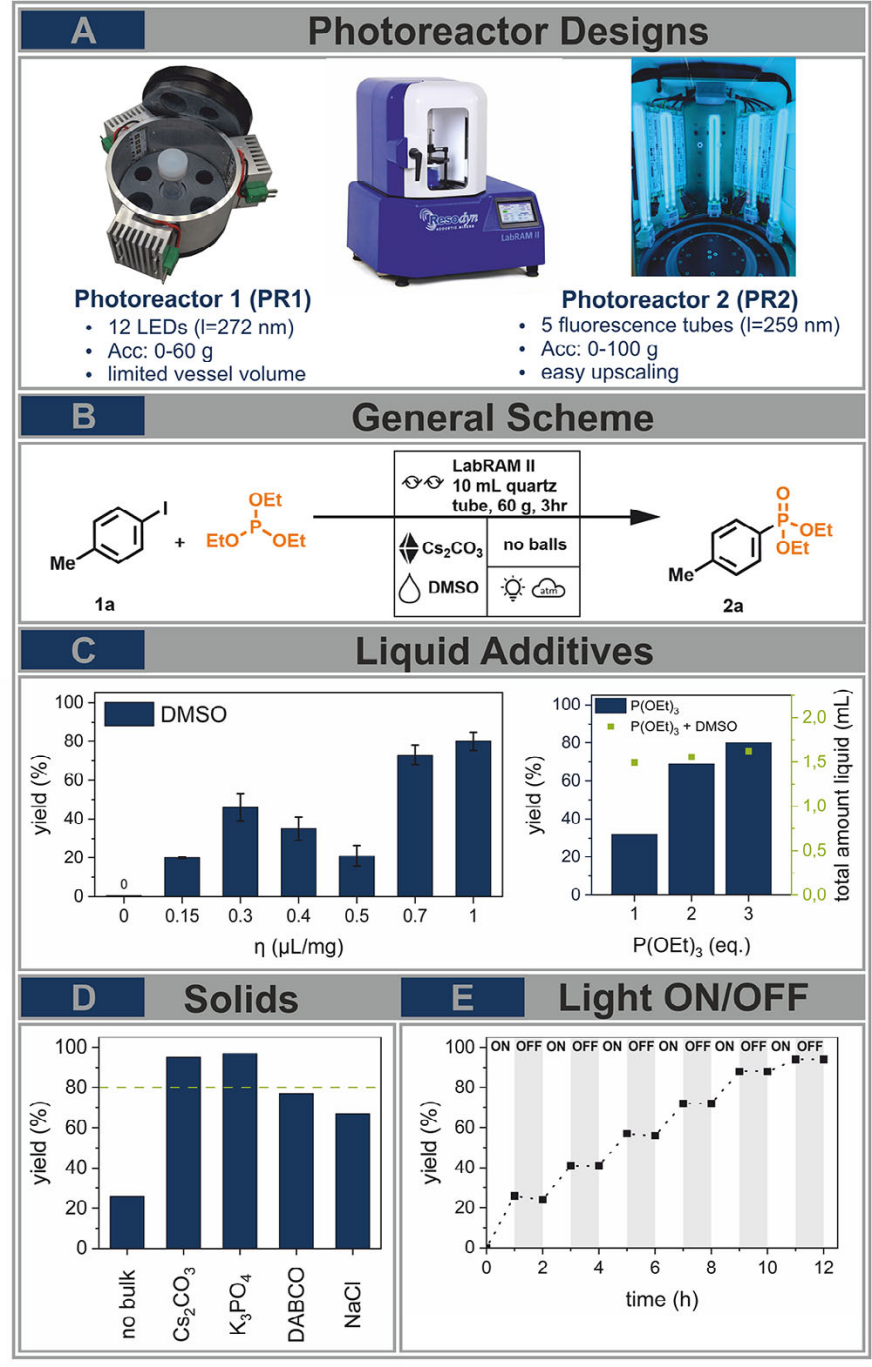
A) Photoreactor designs for the resonant acoustic mixer (RAM, middle), left (**PR1**) based on LED arrays, right (**PR2**) based on low‐pressure mercury fluorescence tubes. B) General scheme of model reaction, photochemical phosphonation reaction of 4‐iodotoluene (1a) with triethyl phosphite, reaction conditions if not stated otherwise: 0.38 mmol 1a, 3 eq. P(OEt)_3_, 2 eq. Cs_2_CO_3_, 1 g SiO_2_, and DMSO (η= 0.3) were mixed in a 14 mL quartz glass tube at 60 g in the RAM for 3 hours under ambient air conditions and constant irradiation of 12 UVC‐LEDs (*λ*= 272 nm) in **PR1**. C) Investigation of the influence of liquid additives including, left amount (η) of DMSO and right equivalents of P(OEt)_3_ in dependency of the total amount of liquid using DMSO with η=1. D) Influence of solid additives, green line indicates the yield under previously optimized conditions. E) Time‐dependent light on/off experiment of the phosphonation reaction of 4‐iodotoluene (1eq.) under optimized conditions mixing 3 eq. P(OEt)_3_, 15 eq. K_3_PO_4_, DMSO (η=1) under ambient air conditions in the RAM at 60 g for 1 hour under irradiation using **PR1** followed by 1 hour in the dark. The process was repeated until full conversion of the substrate to the product was achieved. Corresponding conversions were determined by ^1^H‐NMR spectroscopy by analyzing a small amount of the crude reaction mixture.

Since previous investigations on photochemical reactions in the ball mill revealed that the mechanical impacts by the milling balls play no crucial role in the activation of the substrates but instead ensures sufficient mixing and irradiation of the powder, we implemented the application of photochemistry inside a RAM on the example of the C─P functionalization of aryl halides which is an important transformations in synthetic organic chemistry playing a crucial role in catalysis, medicinal chemistry, agriculture, and material science. ^[^ ^21a–e^ ^]^ To realize this new photochemical approach inside the RAM we optimized the reaction parameters focusing on liquid and solid additives to achieve superior yields. Furthermore, we developed two photoreactor designs and compared their robustness, efficiency, and applicability inside the RAM, elaborating the advantages and limitations of such designs to be able to perform feasible upscaling (Figure 1A).

## 2 Results and Discussion

### 2.1 Influence of Additives

As a model reaction we chose the light‐driven phosphonation reaction of aryl halides which has previously been studied in a ball mill.^[^ ^9^ ^]^ We designed a custom cylindrical photoreactor (**PR1,** Figure 1A, left) consisting of two polyoxymethylene (POM) vessel holder plates and an aluminium ring housing three UV light emitting diode (LED) arrays. To avoid overheating of the LED arrays, three external fans were arranged around **PR1** (Figure S2). Because the rheology of the reaction mixture is crucial for the outcome of the mechanochemical reaction performed inside the RAM,^[^ ^14^ ^]^ we started by investigating the influence of the liquid additives (Figure 1C). As initial reaction conditions we mixed 0.38 mmol 4‐iodotoluene (**1a**), 3 eq. of triethyl phosphite (P(OEt)_3_), and 2 eq. of caesium carbonate with 1 g of silica as a bulk material and the corresponding amount of DMSO as LAG agent at 60 g for 3 hours under ambient conditions inside a custom‐made quartz glass reaction vessel (Figure 1B). Next to neat conditions (η = 0), we tested η‐values between 0.15 and 1. No yield was observed under neat conditions, emphasizing the need for a liquid to facilitate molecular‐level mixing. Besides that, we observed two η‐sweet spots at 0.3 and 1 µL/mg. At η = 0.3, the paste‐like rheology led to a local maximum of 46% yield of the desired product (**2a**) according to ^1^H‐nuclear magnetic resonance (NMR) spectroscopy using dibromo methane as an internal standard. The product formation was the same under inert conditions, demonstrating the robustness of the reaction against ambient air. At η = 0.4 and 0.5, the rheology properties of the reaction mixtures deteriorated further leading to decreased yields, since the sticky consistency caused agglomeration at the bottom of the vessel, resulting in insufficient mixing and reproducibility (Figure S5). Only from η = 0.7, the rheology allowed unhindered mixing showing the best result at η = 1 yielding 80% of product **2a**, according to ^1^H‐NMR spectroscopy. We continued our further investigations at η = 1, examining decreased amounts of the liquid phosphonation agent triethyl phosphite of 1 and 2 equivalents observing decreased yields of 37% and 69%, respectively (Figure 1C, right). To assess whether the diminished product formation is attributable to reduced accessibility of the phosphonating agent or to rheological changes resulting from a proportional decrease in the total liquid volume (Figure 1C, right, green dots), we increased the amount of DMSO while maintaining 1 equivalent of P(OEt)_3_. This adjustment was intended to replicate the total liquid volume used in the reaction with 3 equivalents of P(OEt)_3_. Since the yield stayed low (46%, Table S2, Entry 4), the lower amount of P(OEt)_3_ is the cause of the reduced reactivity.

In addition to the influence of liquid additives, we turned toward solid additives. In previous ball‐milling experiments silica was used as an inert milling auxiliary to ensure a powdery non‐agglomerated mixture to minimize mechanical impacts from ball‐vessel wall interactions. The RAM set‐up, which avoids milling balls, benefits from a more slurry‐like rheology to enhance mixing and substrate contacts. Therefore, we reevaluated the need for silica and substituted it by a mass equivalent (1 g) of the corresponding base caesium carbonate (10 eq.) observing an increased yield of 93% (Figure 1D). Other bases, like potassium phosphate (15 eq.), a common base in mechanochemical approaches, and 1,4‐diazabicyclo [2.2.2] octane (DABCO) (25 eq.), a commonly used base in solution‐based phosphonation reactions yielded **2a** in 93% and 77%, respectively (since previous investigations in the ball mill showed low to no improvement when testing other bases,^[^ ^9^ ^]^ we omitted further investigations at this point). When we omitted the addition of any solid additives, the yield decreased to 23%, which relates to the insufficient mixing and irradiation due to poor rheology. To examine if the decrease is additionally related to the absence of a base, we performed a reference experiment with sodium chloride as a neutral solid additive, observing 68% of product **2a**. For this reason, we believe that a moderately strong base like carbonates or phosphates facilitates the product formation by stabilizing the photo‐activated intermediate.^[^ ^22^ ^]^ Regarding the cost efficiency, we established our optimized reaction conditions using 15 eq. of K_3_PO_4_ as the bulk and base.

Having optimized the reaction conditions and investigated the influence of additives, we next turned our attention to the time‐resolved photochemical nature of the reaction. A light on/off experiment conducted under continuous mixing further confirmed the photochemical character of the transformation: product formation occurred exclusively under irradiation, while mixing in the absence of light resulted in no additional conversion (Figure 1E). Yet, the reaction rate decreased, which may be related to the iterative opening of the milling vessel and sample collection during the ex‐situ monitoring of the reaction progress slowing down the reaction rate due to alterations in the equilibrium.^[^ ^23^ ^]^

**Figure 2:**
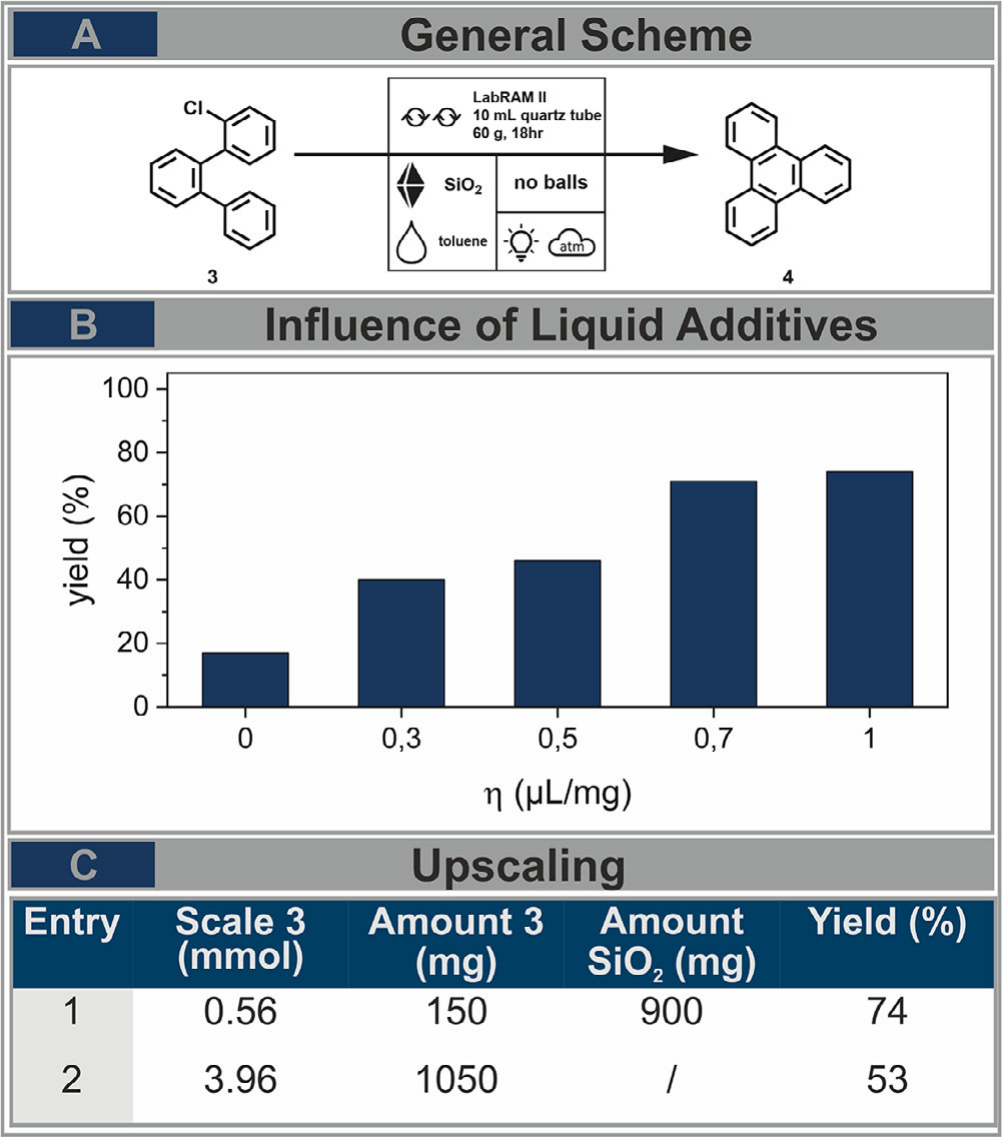
A) Cyclodehydrochlorination reaction of o‐(2‐chloro)‐terphenyl toward triphenylene, reaction conditions as follows: 0.5 mmol 3, 0.9 g SiO2, and toluene (η=0.5) were mixed under ambient air conditions at 60 g for 18 hours under constant irradiation of **PR1**. B) Investigation of the influence of liquid additives using different amounts (η) of toluene. C) sevenfold upscaling experiment of photochemical CDHC reaction under adjustment of reaction conditions (amount of silica as bulk material).

To demonstrate the versatility of reactions feasible under RAM conditions, we decided to further investigate the cyclodehydrochlorination, a photochemical cyclization reaction, from o‐(2‐chloro) terphenyl (**3**) to triphenylene (**4**) (Figure 2A), which has previously been studied under ball milling conditions.^[^ ^8^ ^]^ We started by mixing 150 mg (0.56 mmol) of **3** with 0.9 g silica and η = 0.5 toluene inside a 14 mL quartz glass vessel at 60 g for 18 hours using **P1** yielding 49% triphenylene (**4**) under ambient conditions and 53% under N_2_ atmosphere. Owing to the small deviation, we decided to perform further reactions under air to simplify the preparation procedure. Based on our previous investigations on photochemical reactions in the RAM, we assumed that the amount of liquid additive will have the highest impact on the reaction (Figure 2B). Therefore, we performed reactions with η‐values ranging from 0 to 1 µL/mg. Figure 2B shows the proportional dependency of the reaction outcome on the added amount of liquid, yielding 74% of triphenylene using η = 1 within 18 hours of reaction time. Compared to analogous solution‐based synthesis routes, our method requires only one‐fiftieth of the solvent volume, significantly enhancing its sustainability. Building on our earlier findings from the phosphonation reaction—where silica was found to be nonessential as a bulk material—we scaled up the cyclization reaction sevenfold (3.6 mmol, 1050 mg) in the absence of silica, maintaining a constant total mass in the reaction vessel. This scale‐up yielded 53% of product **4** after 18 hours in **PR1** at 60 g (Figure 2C).

### 2.2 Comparison of Photoreactor Designs

**Figure 3:**
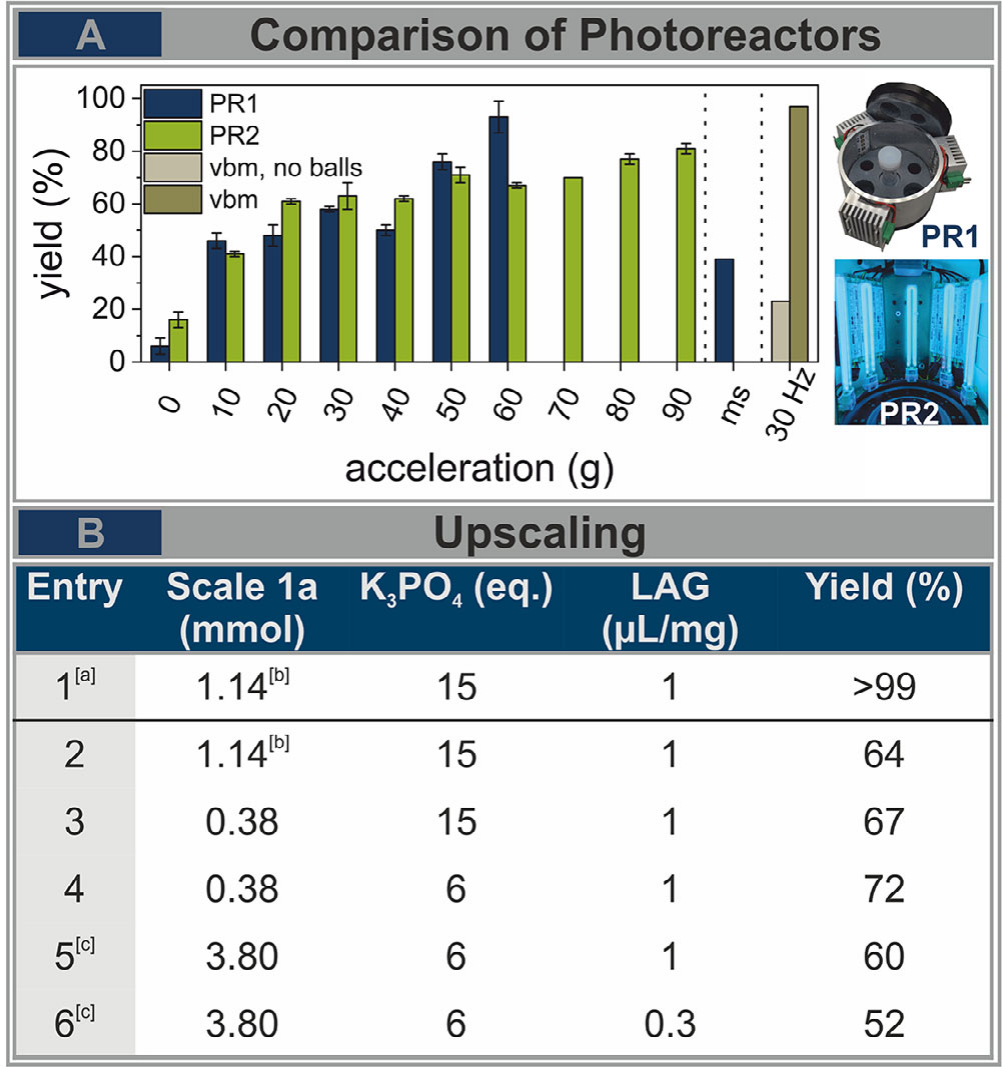
A) Comparison of the photoreactors **PR1** and **PR2** under optimized conditions according to the acceleration of the reaction vessel. **PR1** is limited to accelerations between 0 and 60 g due to its weight. Using **PR2** enabled unlimited accelerations of up to 90 g. Additional comparison to solution‐base conditions (ms: magnetic stirring) using **PR1** and to vibrational ball mill (vbm) at 30 Hz (beige: without milling balls, brown: in addition of 18 PTFE milling balls (d = 5 mm)) under irradiation at *λ*= 254 nm. B) Upscaling experiments of photochemical phosphonation reaction in **PR2** under adjustment of reaction conditions (amount of base and LAG). [a] performed in **PR1**. [b] performed using three normal scale reactions (0.38 mmol) at the same time. [c] performed with a 10‐fold scale in custom quartz‐glass tubes doubled in length (11 cm).

Next to our presented photoreactor design based on UVC LEDs (**PR1**), we developed a second photoreactor based on low‐pressure mercury lamps (**PR2**) (Figure 1A, right). **PR2** consists of six low‐pressure Hg lamps that are steadily mounted on an outer frame (distance vessel‐light source: 13 cm) (Figure S3). Thus, the lamps and the cables experience no movement, which avoids the risk of cable breakage and opens the possibility of performing accelerations between 0 and 90 g, while **PR1** was limited to a maximum acceleration of 60 g due to its weight (it is important to note, that this limitation is specific to the equipment used in our study with the maximum load capacity of 1 kg of LabRAM II and not a general limitation). Additionally, the design and construction of **PR1** required a specialized tool shop. **PR2** can be assembled quite easily from commercial fluorescent tubes and adapters, thus offering a low barrier of entry. To compare both photoreactors, we calculated the theoretical number of emitted photons for **PR1** and **PR2** to 7.70 and 6.64*10^−8^mol/s, respectively (see Supporting Information). Additionally, the temperature development was monitored inside both photoreactors (Figure S4). We continued investigating the influence of higher accelerations on the model reaction toward **2a** (photochemical phosphonation) under the optimized reaction conditions (Figure 3A). Without mixing (0 g), both photoreactors showed low yields of 6% (**PR1**) and 16% (**PR2**). Notably, at higher accelerations, **PR1** showed a continuous increase in product formation, whereas reactions performed with **PR2** were only marginally affected. The difference likely arises from the altered geometries of the light sources. Owing to the larger distance between **PR2** and the reaction vessel as well as the total length of the fluorescent tubes the number of deflected photons increased. However, **PR2** is more efficient at accelerations below 50 g compared to **PR1**. To compare the efficiency of resonant acoustic mixing with that of conventional mechanochemical as well as solution‐based synthesis, we also performed references experiments. First, we prepared a reaction under optimized conditions in absence of milling media and mixed it at 30 Hz under irradiation (*λ* = 254 nm) for 3 hours observing only 23% yield (Figure 3A, beige). In contrast, the same reaction conditions under addition of 18 PTFE milling balls (d = 5 mm) showed almost full conversion (97%, Figure 3A, brown). These experiments show that milling balls are essential to achieve sufficient mixing in a ball mill. Further they emphasize the superior mixing power of the RAM that operates in complete absence of additional milling media. Finally, we invested the solution‐based conditions by adding a magnetic stirring bar and plate inside **PR1** (Figure 3A, ms = magnetic stirring), which yielded only 39%. This result underlines again the high efficiency of mixing powders in the RAM compared with conventional stirring in solution‐based approaches.

### 2.3 Upscaling

In contrast to ball mills, RAM offers a wide range of options for easy upscaling. **PR1** enables up to four vessel positions, one in the center and three outer positions, one in front of each LED array (distance LED‐vessel: 0.9 cm). A straightforward threefold scale‐up was achieved by preparing three identical reactions under optimized conditions and processing them simultaneously in **PR1**. After 3 hours of irradiation, the reaction mixtures were combined and purified, affording the product in > 99% yield as determined by ^1^H‐NMR spectroscopy (Figure 3B, Entry 1). A comparable strategy applied in **PR2**—running three standard reactions concurrently—resulted in a 64% yield, closely matching that of the standard‐scale reaction (67%, Figure 3B, Entries 2 and 3, respectively).

Additionally, the design of **PR2** enables extension of the quartz glass tube length from 5 cm to 11 cm (Figure S1), offering an alternative route for reaction scale‐up. To achieve a tenfold increase in scale—from 0.38 mmol to 3.8 mmol—it was necessary to adjust the reaction conditions by reducing the total powder volume, specifically by lowering the amount of K₃PO₄ from 15 to 6 equivalents (Figure 3B, Entry 5). Under these conditions, a 60% yield was obtained, compared to 72% in a standard‐scale reference reaction (Figure 3B, Entry 4), while still using only a fraction of the solvent typically required in solution‐based protocols. Further reduction of the DMSO content to η  =  0.3 led to a decreased yield of 52%, likely due to altered rheological properties affecting reaction efficiency. Nevertheless, we believe that RAM technology holds significant potential for enhancing the performance and scalability of photochemical reactions. By reducing reaction time and solvent usage, it offers a high‐throughput, energy‐efficient, and sustainable alternative for advancing solid‐state photochemistry.

## 3 Conclusion

To the best of our knowledge, we demonstrated the first successful combination of photochemistry and resonant acoustic mixing in the absence of milling media, exemplified by two distinct photochemical reactions – the phosphonation of aryl halides and the CDHC. We investigated the influence of liquid and solid additives to optimize the rheology and examine the impact on product formation. We observed two sweet spots (η = 0.3 and 1) that showed favorable rheological conditions. Owing the reproducibility and higher yield, we continued with an η‐value of 1. We tested the influence of lower equivalents of the phosphonating agent and clarified its role in regards of rheology and the reaction mechanism. Further, we investigated the influence of solid additives screening different solid bases as bulk materials. Here, the replacement of silica by an equivalent mass of K_3_PO_4_ increased the product formation toward 97% yield performing as good as Cs_2_CO_3_. Yet, the former base convinced due to the lower costs. In the next step, we examined the reaction kinetics by performing a reaction under oscillating irradiation and in the absence of light, observing the true photochemical character of the transformation. We transferred the method to another photochemical reaction examining the regioselective ring closure by C─Cl bond activation, forming triphenylene. As expected, we observed a significant influence by increasing the amount of the liquid additive giving 74% triphenylene with an η‐value of 1. By introducing a second photoreactor design based on fluorescence tubes, we compared the effects of different light sources and their geometries, as well as the influence of different accelerations. This opened the possibility of increasing not only the number of reaction vessels from one to three but also the vessel volume and performing successful upscaling experiment up to a 10‐fold scale. The implementation of photochemical reactions in the field of solid‐state chemistry using RAM opens a completely new field in photochemistry under the active reduction of solvents without the penalties caused by poor diffusion and light penetration. Furthermore, it may have significant implications regarding the easy upscaling of photochemical solid‐state reactions.

## Conflict of Interest

The authors declare no conflict of interest.

## Supporting information



Supporting Information

## Data Availability

The data that support the findings of this study are available in the supplementary material of this article.
