# Supplementary material for: Solid‐State Photochemistry in Resonant Acoustic Mixers
Source: Chemistry. 2025 Jun 12;31(38):e202501137. doi: 10.1002/chem.202501137 (PMC12238916; doi:10.1002/chem.202501137)
Supplement: Supplementary file 1 — Supporting Information [file CHEM-31-e202501137-s001.pdf]

Supporting Information

Solid-State Photochemistry in Resonant Acoustic Mixers

Carolina Spula,[a] Phil M. Preuß,[a],Lars Borchardt [a] and Sven Grätz*[a]

[a] C. Spula, P. M. Preuß, Prof. Dr. L. Borchardt, Dr. S. Grätz
Inorganic Chemistry I
Ruhr-Universität Bochum
Universitätsstr. 150, 44801 Bochum (Germany)
E-mail: sven.graetz@rub.de

Table of Contents

EXPERIMENTAL SECTION 2

1.1. General information 2

1.2. Photoreactor designs 3
1.2.1. Transparent reaction vessels 3
1.2.2. UVC-LED Photoreactor (PR1) 4
1.2.3. UV-Fluorescence tubes (PR2) 5

1.3. Comparison of Photoreactors 6
1.3.1. Calculation of number of emitted photons per time unit for PR1 6
1.3.2. Calculation of number of emitted photons per time unit for PR2 6
1.3.3. Temperature measurements 6

2. EVALUATION OF REACTION PARAMETER 8

2.1. Photochemical solid-state phosphonation reaction 8
2.1.1. Investigation on liquid additives 8
2.1.2. Investigation of solid additives 9
2.1.3. Radical trapping experiment 11
2.1.4. Radical iodine clock experiment 11
2.1.5. Time Screening 11
2.1.6. Light on/off experiment 12
2.1.7. Acceleration Screening 13
2.1.8. Up-Scaling 14

2.2. Photochemical solid-state CDHC reaction 16
2.2.1. Influence of liquid additives 16
2.2.2. Up-Scaling 16

3. SYNTHETIC PROCEDURES 17

3.1. General procedure for photochemical phosphonation reaction in the RAM (G1) 17

3.2. General procedure for photochemical CDHC reaction in the RAM (G2) 17

4. CHARACTERIZATION 19

5. NUCLEAR MAGNETIC RESONANCE SPECTRA 21

6. REFERENCES 28

Experimental Section

1.1. General information

General All reagents were obtained from commercial suppliers at least in synthesis grade purity and were used without further purification. Organic solvents used for LAG were obtained in analysis grade. The milling was carried out in a *LabRAM II* resonant acoustic mixer purchased from Resodyn Acoustic Mixers Inc. The milling vessels consist of quartz glass tubes capped with custom-made PFA caps.

^{1}H nuclear magnetic resonance (^{1}H-NMR), **^{13}C nuclear magnetic resonance** (^{13}C-NMR), and **^{31}P nuclear magnetic resonance** (^{31}P-NMR) spectra were recorded in $CDCl_3$ on a *Bruker Avance III HD* spectrometer. The ^{1}H-NMR and ^{13}C-NMR spectra were referenced internally to residual solvent resonances and are reported relative to tetramethylsilane. Chemical shifts are reported in ppm from tetramethylsilane. Data are reported as follows: s = singlet, d = doublet, dd = doublet of doublet, m = multiplet; coupling constants in Hertz.

Photoreactors: For PR1, the light emitting diode (LED)-based setup for the photochemical irradiation, High Power UV-C-LEDs purchased from *Laser Components GmbH* were used. In total, three LED units consisting of 4 LED elements each**,** were built into a custom-made aluminum cylinder. The LED units emitted with a wavelength of λ = 272 nm and had a power of P_{LED} = 0.4 Js respectively. The quartz glass vessels were secured by two polyoxymethylene plates on top and bottom of the aluminum ring. The LEDs were cooled actively by three ventilators.

For PR2, the Hg lamps photoreactor, 5 low-pressure mercury-containing fluorescence lamps (*OSRAM* Puritec HNS L 24W, 30 cm long) were mounted on carrier plate made from aluminium which was screwed to the outer corpus of the reaction chamber to form a half spherical shape. They emit at a wavelength of 254 nm.

Temperature measurements: To determine the internal temperature of milling vessels, an *iButton DS1923-F5#* temperature logger was used to track the temperature evolvement during the reaction.

Flash Chromatography for purification of the products was performed using an *Advion Interchim scientific puriFlash 5.020* equipped with an UV detector as well as an ELSD detector.

1.2. Photoreactor designs

In the following paragraph, we present and compare various light sources, including the UVC LED photoreactor PR1 and low-pressure mercury (Hg) lamps PR2 with different emission wavelengths.

1.2.1. Transparent reaction vessels

All reactions were performed in UV-C transparent custom-made reaction vessels consisting of a quartz-glass tube enclosed by two polyfluoroalkyl (PFA) lids (**Figure S1**). Parameter optimizations were performed in standard size (left). For upscaling reactions doubled length tubes were used (right).

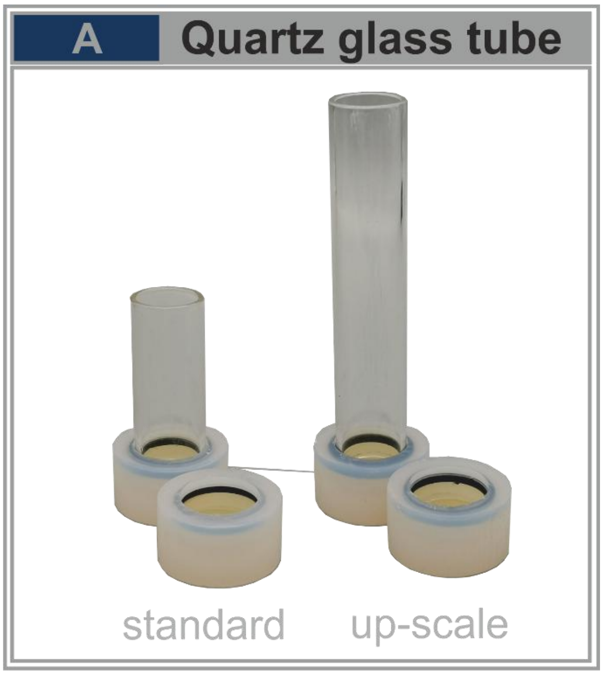

Figure S1. Reaction vessels based on quartz glass tubes closed by two polyfluoroalkyl (PFA) lids in standard size (5 cm, left) and up-scaled size (11 cm, right).

1.2.2. UVC-LED Photoreactor (PR1)

The photoreactor based on UVC LEDs (**PR1**) consists of three parts forming a cylinder- an aluminum ring (d_{outer}=130 mm, d_{inner}= 126 mm, h=65 mm) bearing 3x4 LED arrays (λ= 272 nm) located with equal distance (120°) to each other and two polyoxymethylene (POM) vessel holders (d=130 mm, h=27 mm) that secure the reaction vessel in place and close the aluminum ring on top and bottom. The reaction vessel is placed in a central vessel holder position with an equal distance of 44 mm to all three LED arrays. The aluminum ring is equipped with cooling rips which are externally cooled by three ventilations. The LEDs can be controlled individually, yet, if not stated otherwise, all reactions were performed using 3x4 LEDs at a time.

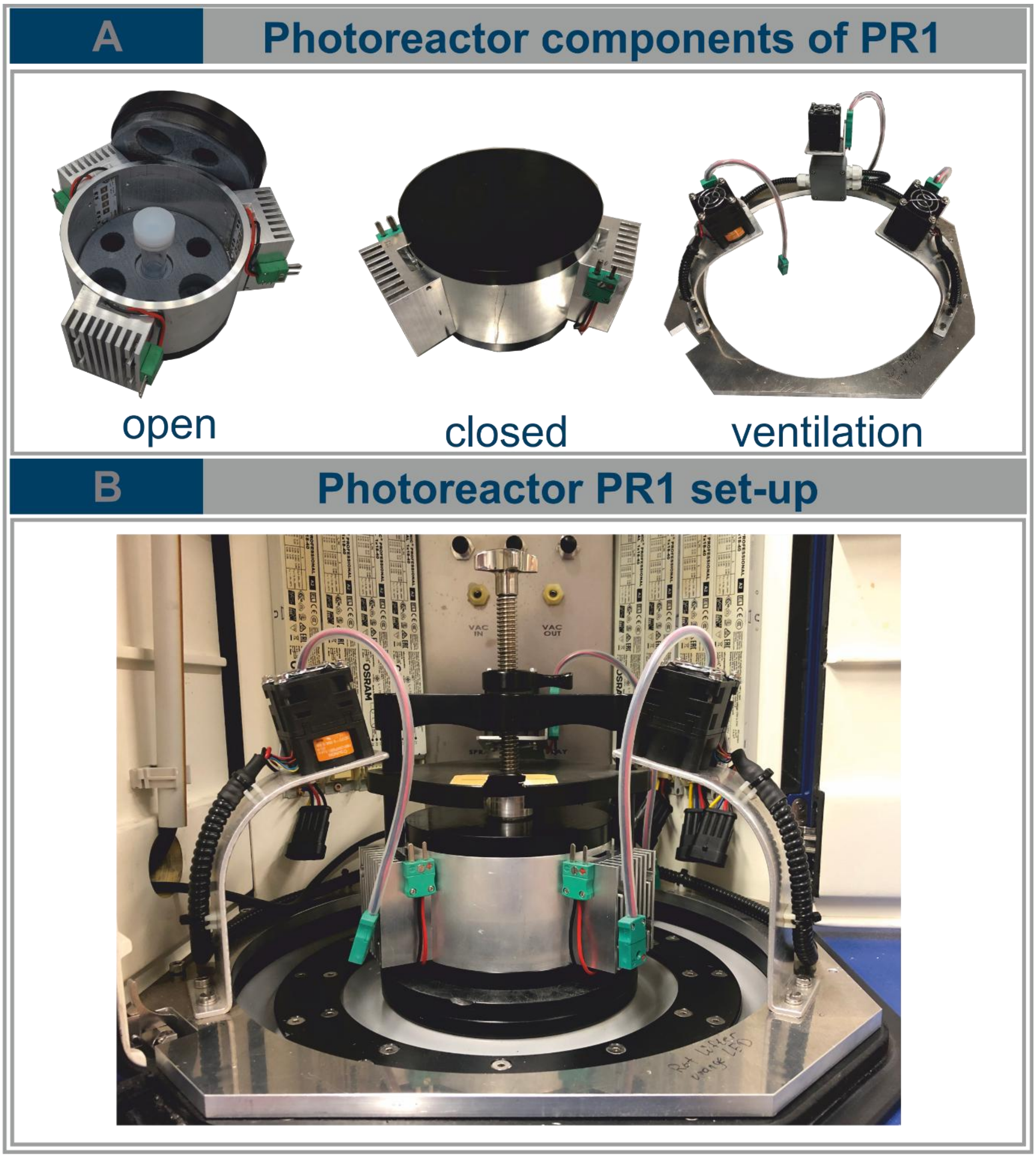

Figure S2: Design and construction of photoreactor 1 (PR1) based on LED arrays. A: Components of PR1. B: Complete set-up of PR1 inside the RAM.

1.2.3. UV-Fluorescence tubes (PR2)

The photoreactor based on low pressure mercury lamps (**PR2**) consists of an aluminum ring (d_{inner}=310 mm) bearing five low pressure mercury lamps (l=x mm, h=x mm) arranged in a semi-circle (d_{inner}=290 mm). The aluminum ring is screwed to the outer plate of the mixing chamber and therefore is not affected by the acceleration of the vibration plates.

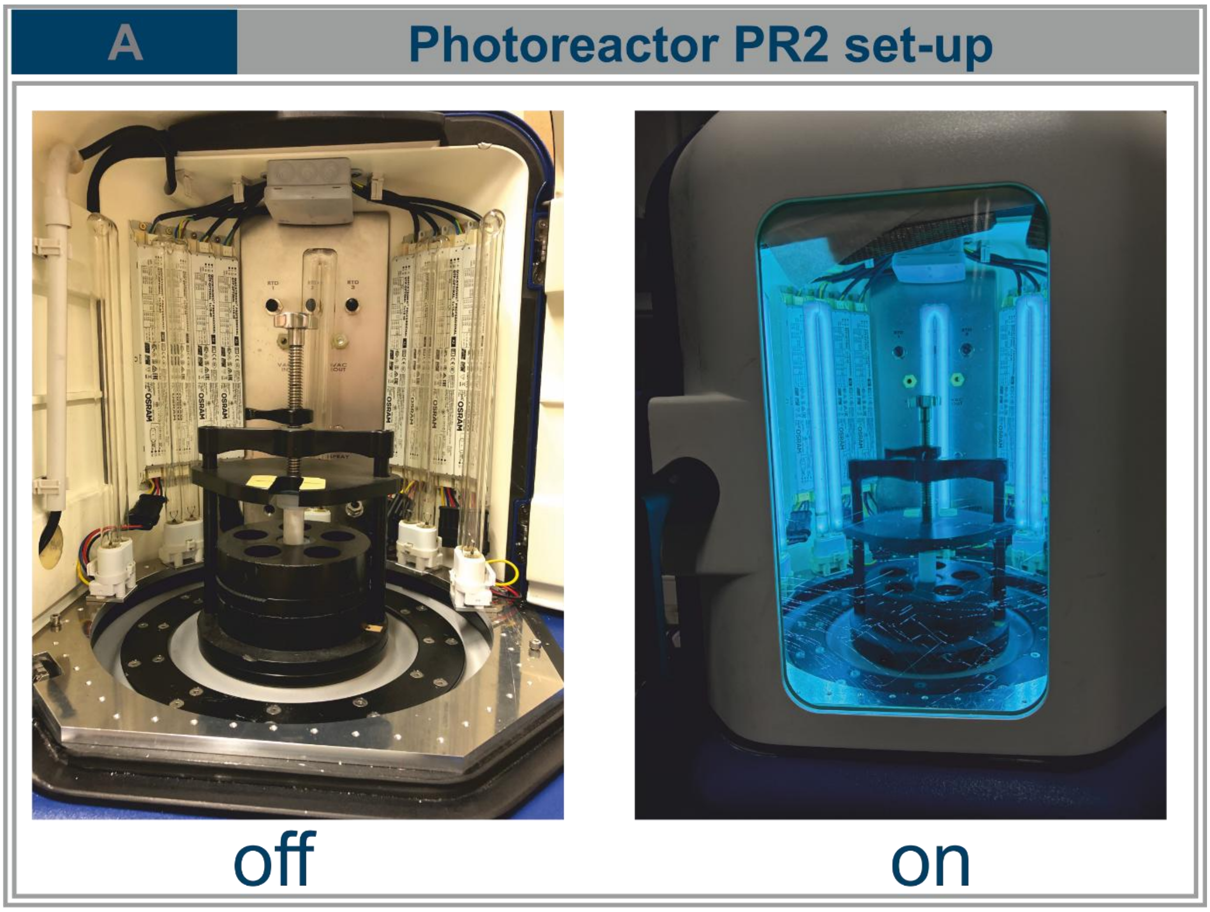

Figure S3: Set-up of photoreactor 2 (PR2) based on fluorescent tubes turned off and on for better visualization. The window was covered during the reaction.

1.3. Comparison of Photoreactors

1.3.1. Calculation of number of emitted photons per time unit for PR1

$$E_{272nm} = h \cdot \frac{c}{\lambda} = 6{,}6 \cdot 10^{-34} Js \cdot \frac{3 \cdot 10^{8}\ \frac{m}{s}}{272 \cdot 10^{-9}\ m} = 7.28 \cdot 10^{-19}\ J$$

Equation 1: Calculation of energy E of one photon emitted at a wavelength of λ=272 nm with c as speed of light and h as planck constant.

$$n(Photons)_{PR1} = \frac{P_{LED} \cdot V_{vessel}}{E \cdot N_A} = \frac{22.5\ \frac{W}{m^2} \cdot 0.0015\ m^2}{7.28 \cdot 10^{-19}\ J \cdot 6.02 \cdot 10^{23}\ \frac{1}{mol}} = 7.70 \cdot 10^{-8}\ \frac{mol}{s}$$

Equation 2: Calculation of number n of photons emitted by PR1 with P as radiance, V as volume of reaction vessel, E being the energy of one single photon emitted as a wavelength λ of 272 nm and N_A as the Avogadro constant.

1.3.2. Calculation of number of emitted photons per time unit for PR2

$$E_{254nm} = h \cdot \frac{c}{\lambda} = 6{,}6 \cdot 10^{-34} Js \cdot \frac{3 \cdot 10^{8}\ \frac{m}{s}}{254 \cdot 10^{-9}\ m} = 7.80 \cdot 10^{-19}\ J$$

Equation 3: Calculation of energy E of one photon emitted at a wavelength of λ=254 nm with c as speed of light and h as planck constant.

$$n(Photons)_{PR2} = \frac{P_{Hg} \cdot V_{vessel}}{E \cdot N_A} = \frac{20.8\ \frac{W}{m^2} \cdot 0.0015\ m^2}{7.80 \cdot 10^{-19}\ J \cdot 6.02 \cdot 10^{23}\ \frac{1}{mol}} = 6.64 \cdot 10^{-8}\ \frac{mol}{s}$$

Equation 4: Calculation of number n of photons emitted by PR1 with P as radiance, V as volume of reaction vessel, E being the energy of one single photon emitted as a wavelength λ of 254 nm and N_A as the Avogadro constant.

1.3.3. Temperature measurements

We monitored the temperature development of the reaction vessel during irradiation. Therefore, we tracked both photoreactors over independent three-hour reactions with an *iButton* temperature logger attached close to the vessel. The temperatures in PR1 and PR2 increased to 55°C and 51°C, respectively.

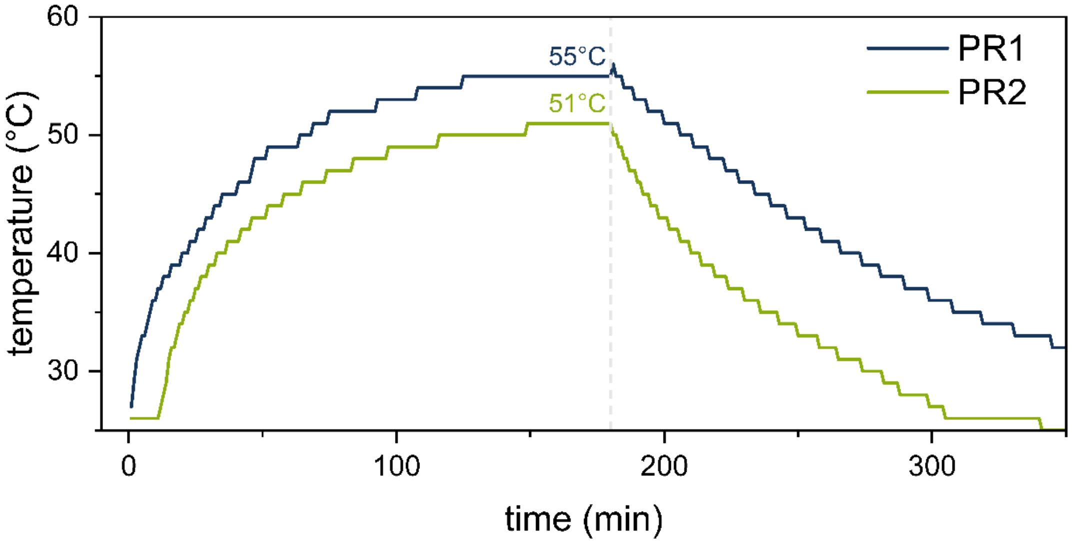

Figure S 4: Temperature profiles of PR1 (blue) und PR2 (green) over time at n acceleration of 60 g in the RAM. The end. The end of the reaction (180 min) is indicated by the grey dashed line.

2. Evaluation of reaction parameter

2.1. Photochemical solid-state phosphonation reaction

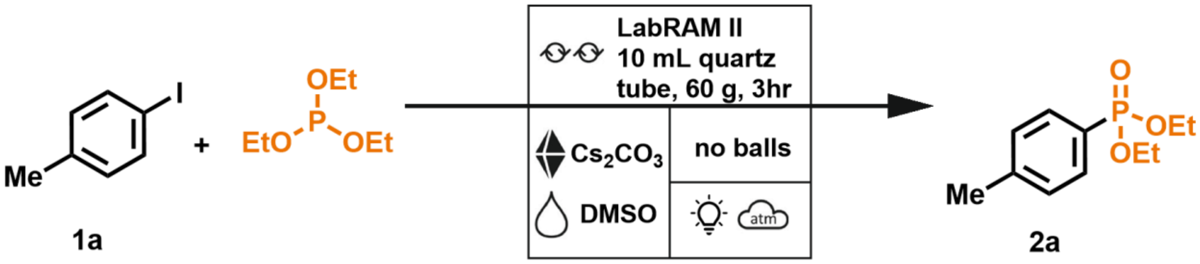

Scheme S1: Photochemical phosphonation reaction of 4-iodo toluene with $P(OEt)_3$ in the RAM.

2.1.1. Investigation on liquid additives

We started our investigations on the photochemical phosphonation reaction by adjusting the rheology by testing different amounts (η=µL/mg) of the liquid additive DMSO between 0 and 1 µL/mg (Table S1). We determined two η-sweet spots, at 0.3 And 1 µL/mg, with local maxima in yields of 46 and 80%. Yet, the results at η-values between 0.3 and 0.5 µL/mg were not reproducible due to the sticky and paste-like rheology of the reaction mixture, causing the substrates to accumulate in the bottom PFA cap hindering sufficient mixing and irradiation (Figure S3). Above an η-value of 0.7 µL/mg, the rheology was fluid enough to enable adequate mixing.

Table S1: Investigation of different amounts of the liquid additive DMSO with corresponding yields. Each experiment was performed using 83 mg of 4-iodotoluene, 3 eq. $P(OEt)_3$, 1 g silica and 2 eq. of Cs_2CO_3 which were mixed together with the corresponding amount of DMSO inside a 10 mL quartz glass tube enclosed by two PFA caps in the RAM at 60 g for 3 h under constant irradiation of 12 LEDs (λ=272 nm) inside the PR1. Yields were determined according to ^{1}H-NMR spectroscopy using dibromo methane as an internal standard.

Entry	η (µL/mg)	yield (%)
1	0	0.5 ± 0
2	0.15	20 ± 0.5
3	0.3	46 ± 7
4	0.4	35 ± 0
5	0.5	21 ± 5
6	0.7	73 ± 5
7	1	80 ± 5

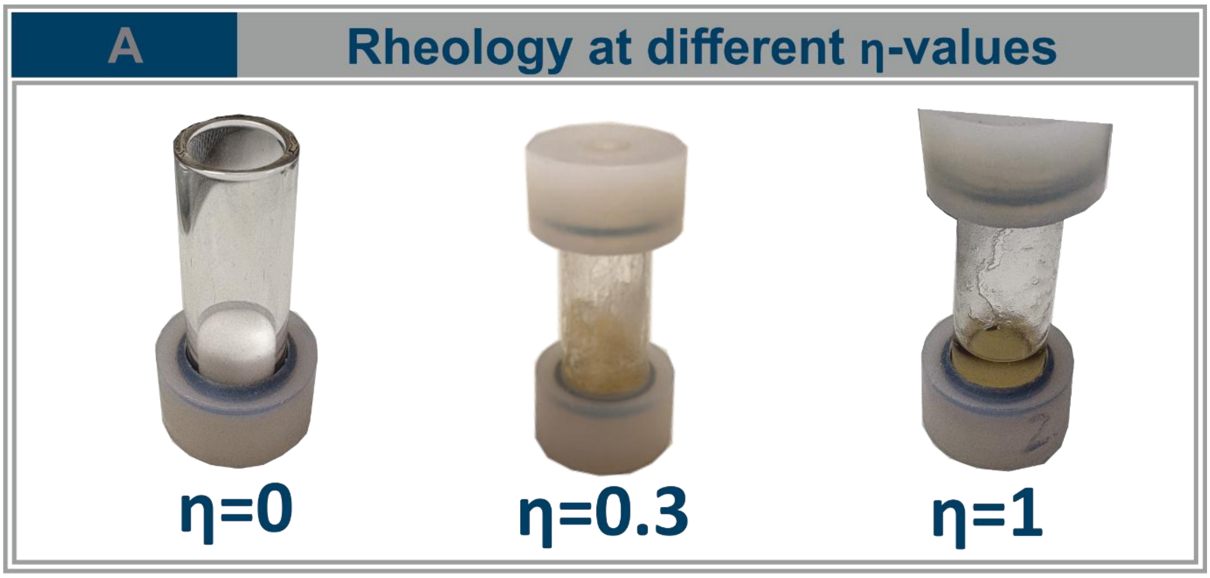

Figure S5: Rheology of the reaction mixture after the reaction time at corresponding η-values of DMSO.

By optimizing the rheology by increasing the amount of DMSO, we investigated the effect of decreasing amounts of the liquid phosphonating agent $P(OEt)_3$. Therefore, we performed reactions using 1 and 2 eq. of $P(OEt)_3$ observing a decreasing product formation. Additionally, we performed a reference experiment using 1 eq. of $P(OEt)_3$ but increasing the amount of DMSO to a total amount of liquid equal to the experiment at 3 eq. $P(OEt)_3$ to ensure a similar rheology.

Table S2: Investigation of different amounts of the liquid additive $P(OEt)_3$ with corresponding yields. Each experiment was performed using 83 mg of 4-iodotoluene, 1 g silica and 2°eq. of Cs_2CO_3, 1 µL/mg DMSO and the corresponding amount of $P(OEt)_3$ which were mixed inside a 10 mL quartz glass tube enclosed by two PFA caps in the RAM at 60 g for 3 h under constant irradiation of 12 LEDs (λ=272 nm) inside the PR1. Yields were determined according to ^{1}H-NMR spectroscopy using dibromo methane as an internal standard.

Entry	Amount $P(OEt)_3$ (eq.)	Total amount of liquid (mL)[a]	Yield (%)
1	1	1.396	37 ± 6
2	2	1.462	69 ± 6
3	3	1.528	80 ± 4
4[b]	1	1.528	46
5[c]	1	1.528	39

[a] considering the added volumes of DMSO and $P(OEt)_3$. [b] to equalize the total amount of liquid inside the reaction mixture we increased the amount of DMSO by 130 µL, which corresponds to the difference between 1 (66µL) and 3 eq. (196 µL) of $P(OEt)_3$. [c] experiment performed in the absence of silica and Cs_2CO_3 with the final optimized conditions using 15 eq. K_3PO_4 as bulk and base.

2.1.2. Investigation of solid additives

We performed similar investigations of the solid additives for both η-sweetspots observed during the η-screening (**Table S1**), namely η=0.3 and 1. In both cases, we observed that the absence or replacement of silica by an equivalent amount of base showed constant or increased yields. In the case of η=1, we tested DABCO, a commonly used base in solution-based chemistry, as well as NaCl as a neutral reference additive.

Table S3: Investigation of different types and amounts of the solid additive (base and/or milling auxiliary) with corresponding yields. Each experiment was performed using 83 mg of 4-iodotoluene, 3 eq. $P(OEt)_3$, 0.3 µL/mg DMSO and the corresponding amount of added base and/or silica as a milling auxiliary. The mixture was mixed inside a 10 mL quartz glass tube enclosed by two PFA caps in the RAM at 50 g for 3 h under constant irradiation of 12 LEDs (λ=272 nm) inside the PR1. Yields were determined according to ^{1}H-NMR spectroscopy using dibromo methane as an internal standard.

Entry	Amount SiO_2 (g)	Type Base	Amount base (g)	Yield (%)
1	1	Cs_2CO_3	0.234	43 ± 2
2	/	Cs_2CO_3	1.234	89
3	1	K_3PO_4	0.153	73 ± 2
4	/	K_3PO_4	0.153	71
5	/	K_3PO_4	1.234	74 ± 3

Table S4: Investigation of different types and amounts of the solid additive (base and/or milling auxiliary) with corresponding yields. Each experiment was performed using 83 mg of 4-iodotoluene, 3 eq. $P(OEt)_3$, 1 µL/mg DMSO and the corresponding amount of added base and/or silica as a milling auxiliary. The mixture was mixed inside a 10 mL quartz glass tube enclosed by two PFA caps in the RAM at 50 g for 3 h under constant irradiation of 12 LEDs (λ=272 nm) inside the PR1. Yields were determined according to ^{1}H-NMR spectroscopy using dibromo methane as an internal standard.

Entry	Amount SiO_2 (g)	Type Base	Amount base (g)	Yield (%)
1	No auxiliary	No base	/	23 ±3
2	/	Cs_2CO_3	1.234	93 ± 3
3	/	K_3PO_4	1.153	93 ± 6
4	/	DABCO	1.080	77 ± 1
5	/	NaCl	1.153	68 ± 1

2.1.3. Radical trapping experiment

To investigate the mechanism of the phosphonation reaction, we performed a radical trapping experiment using 2,2,6,6-tetramethypiperidineyloxyl (TEMPO) as a radical trapping agent. Therefore, 83 mg of 4-iodotoluene, 1.2 eq. TEMPO, 3 eq. P(OEt)3, 1 µL/mg DMSO and 1.15 g K_3PO_4 into the quartz glass vessel and mixed at 50 g under constant irradiation in PR1 for 3 h. The reaction was purified by extraction with ethyl acetate and water. The solvent was removed under reduced pressure. The yield was determined by ^{1}H-NMR spectroscopy using dibromo methane as an internal standard giving 17% of 2a.

2.1.4. Radical iodine clock experiment

To detect free iodine and examine the role of the base during our photochemical phosphonation reaction, we performed various text experiments (**Table S5**) in presence and absence of the base as well as the phosphonate agent. After irradiation, a sample of the crude reaction mixture was mixed with an aqueous starch solution. Bluish discoloration (positive test result) indicated the presence of iodine ions in the reaction mixture, which was only observed in the absence of K_3PO_4. For this reason, we assume that under our optimized reaction conditions, the detached iodine radical is scavenged by the base forming KI.

Table S5: Performed iodine-starch test with various reaction samples. If not stated otherwise, reaction conditions as followed: 83 mg of 4-iodotoluene, 3 eq. $P(OEt)_3$, 1 µL/mg DMSO and 15 eq. K_3PO_4 were mixed at 60 g under constant irradiation of PR1 for 3

Entry	K_3PO_4	$P(OEt)_3$	Test result
1	yes	yes	negative
2	yes		negative
3		yes	positive
4			positive

2.1.5. Time Screening

Table S6: Reaction performance was tracked for individual reactions for corresponding times. Reaction conditions as followed: 83 mg of 4-iodotoluene, 3 eq. $P(OEt)_3$, 1 µL/mg DMSO and 15 eq. K_3PO_4 were mixed at 60 g under constant

irradiation of PR1 for corresponding time. The yield was determined by ^1H-NMR spectroscopy using dibromo methane as internal standard.

Entry	Time (h)	Yield (%)
1	1	54
2	2	88
3	3	98
4	6	97

2.1.6. Light on/off experiment

Table S7: Effects of presence and absence of light on the corresponding yields. Therefore, one reaction was prepared by adding 83 mg of 4-iodotoluene, 3 eq. $P(OEt)_3$, 1 µL/mg DMSO and 1.15 g K_3PO_4 into the quartz glass vessel and mixed at 50 g for 1 h under irradiation in PR1. After removing a small amount of the reaction mixture for analysis, the reaction was continued for another hour in the absence of irradiation and analyzed. This procedure was repeated until the reaction was completed.

Entry	Time (h)	Light	Yield (%)
1	1	On	26
2	2	Off	24
3	3	On	41
4	4	Off	41
5	5	On	57
6	6	Off	56
7	7	On	72
8	8	Off	72
9	9	On	88
10	10	Off	88
11	11	On	94
12	12	Off	94

2.1.7. Acceleration Screening

Table S8: Comparison of performance of PR1 and PR2 at different accelerations ranging between 0 and 60 g or 90 g respectively. Therefore individual reactions were performed under the following conditions: 0.38 mmol 4-iodotoluene, 3 eq. $P(OEt)_3$, 15 eq. K_3PO_4 and DMSO (η=1) were mixed at corresponding acceleration in corresponding photoreactor for 3 h. Yields were determined by ^{1}H-NMR spectroscopy using dibromo methane as an internal standard. Vbm= vibrational ball mill.

Entry	Acceleration (g)	**PR1** Yield (%)	**PR2** Yield (%)
1	0	6 ± 3	16 ± 3
2	10	46 ± 4	41 ± 1
3	20	48 ± 1	61 ± 1
4	30	58 ± 1	63 ± 5
5	40	50 ± 2	62 ± 1
6	50	76 ± 2	71 ± 3
7	60	93 ± 6	67 ± 1
8	70	n.d.	70 ± 0
9	80	n.d.	78 ± 2
10	90	n.d.	81 ± 2
11[a]	stirring, 300 rpm	39	
12[b]	vbm, 30 Hz		23
13[b,c]	vbm, 30 Hz		97

[a] PR1 was placed on top of a magnetic stirring plate and the reaction was mixed by a magnetic stirring bar inside the quartz glass tube at 300 rpm. [b] the reaction was performed inside a vibrational ball mill at 30 Hz under constant irradiation (λ=254 nm). [c] addition of 18 PTFE milling balls (diameter= 5 mm).

2.1.8. Up-Scaling

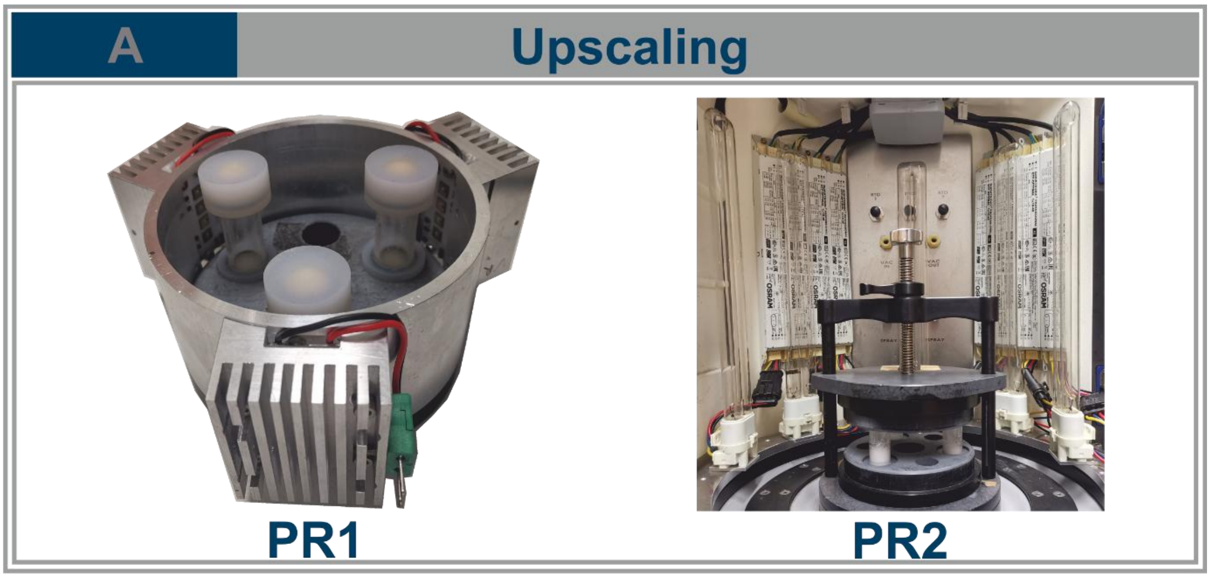

Figure S6: Up-Scaling set up performed in PR1 (left) and PR2 (right). Three normal scale reactions were irradiated at the same time. In the case of PR1 each vessel was placed in front of each LED array to ensure sufficient irradiation.

First, we performed a facile three-fold upscaling experiment in PR1 using three normal scale reactions at the same time (**Figure S4**, left). After the reaction, the three samples were combined and purified, yielding in >99% (Table S9, Entry 2). It is important to note, that the irradiation at the outer positions of the vessel holder are more efficient compared to the central position due to the smaller distance between vessel and LEDs (72% after 1 h of reaction time, Table S9, Entry 1 compared to 54%, Table S6, Entry 1). A similar 3-fold upscaling approach was performed in PR2 (**Figure S4**, right) yielding in 64% **2a** (Table S9, Entry 3). For another up-scaling variation, we used a double-length quartz glass vessel (**Figure S1**) and aimed a ten-times fold reaction scale. Due to the limited volume, we adjusted the amount of base and performed corresponding reference experiments in order to compare the observed yields. First, we decreased the amount of K_3PO_4 to 6 eq under our optimized reaction conditions using the standard quartz glass tubes (Table S9, Entry 5) observing a similar yield of 72% compared to our standard reaction in PR2 (Table S9, Entry 4). We then increased the overall scale of the reaction by a factor of ten (Table 9, Entry 6) observing a s minor decrease towards 60% yield. In an additional experiment, we decreased to amount of DMSO to 0.3 µL/mg which resulted in a decreased yield of 52% (Table 9, Entry 7).

Table S9: Reactions performed to realize ten-fold upscaling reaction using the double-length quartz glass tubes in PR2. Reaction conditions if not stated otherwise: 1 eq. 4-iodotoluene, corresponding amount of K_3PO_4, 3 eq. P(OEt)3, DMSO were mixed in quartz glass reaction vessels for 3 h under constant irradiation of PR2. Yields were determined by ^{1}H-NMR spectroscopy using dibromo methane as internal standard.

Entry	Scale **1a** (mmol)	K_3PO_4 (eq.)	LAG (µL/mg)	Yield (%)
1[a]	0.38[b]	15	1	72[c]
2[a]	1.14[b,d]	15	1	>99
3	1.14[b,d]	15	1	64
4	0.38	15	1	67
5	0.38	6	1	72

6[e]	3.80	6	1	60
7[e]	3.80	6	0.3	52

[a] performed in PR1 [b] vessel placed at outer position of vessel holfer placed directly in front of a LED array. [c] reaction time: 1[d] three normal scale (0.38 mmol) reactions were performed at the same time and purified together. [e] reactions were performed using quartz glass tubes with double length compared to the standard reaction vessel.

2.2. Photochemical solid-state CDHC reaction

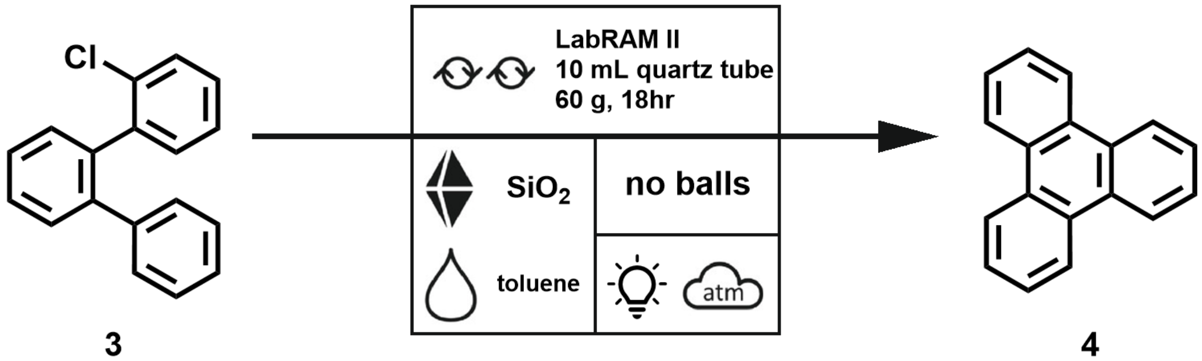

Scheme S 2: Photochemical cyclodehydrochlorination of o-(2-chloro) terphenyl (3) toward triphenylene (4) in the RAM.

2.2.1. Influence of liquid additives

Table 10: Investigation of different amounts η of the liquid additive toluene with corresponding yields. Each experiment was performed using 150 mg of o-(2-chloro) terphenyl (3a) mixed with 0.9 g silica and the corresponding amount of toluene inside a 10 mL quartz glass tube enclosed by two PFA caps in the RAM at 60 g for 18 h under constant irradiation of 12 LEDs (λ=272 nm) inside the PR1. Yields were determined according to ^{1}H-NMR spectroscopy using dibromo methane as an internal standard.

Entry	η (µL/mg)	Yield (%)
1	0	17
2	0.15	40
3	0.3	46
4	0.7	70
5	1	74

2.2.2. Up-Scaling

For the up-scaling reaction we used the standard quartz glass vessels and avoided the use of silica as a bulk material. Therefore, we increased the amount of the substrate **3a** to 1050 mg (Table S11, Entry 2) to ensure the same reactants mass in the reaction vessel as in the standard approach (Table S11, Entry 1) observing a decrease in yield to 53%. It is important to note that the amount of toluene remains the same (η= 1050 µL) while the ratio between 3a and the liquid changes from 17 to 2.5 eq.

Table 11: Upscale approach compared to standard reaction. The corresponding amount of o-(2-chloro) terphenyl (3a) was mixed with the corresponding amount of silica and 1050 µL toluene (η=1) inside a 10 mL quartz glass tube enclosed by two PFA caps in the RAM at 60 g for 18 h under constant irradiation of 12 LEDs (λ=272 nm) inside the PR1. Yields were determined according to ^{1}H-NMR spectroscopy using dibromo methane as an internal standard.

Entry	Scale **3a** (mmol)	Scale **3a** (g)	SiO_2(g)	Yield (%)
1	0.56	0.15	0.9	74
2	3.86	1.05	/	53

3. Synthetic Procedures

3.1. General procedure for photochemical phosphonation reaction in the RAM (G1)

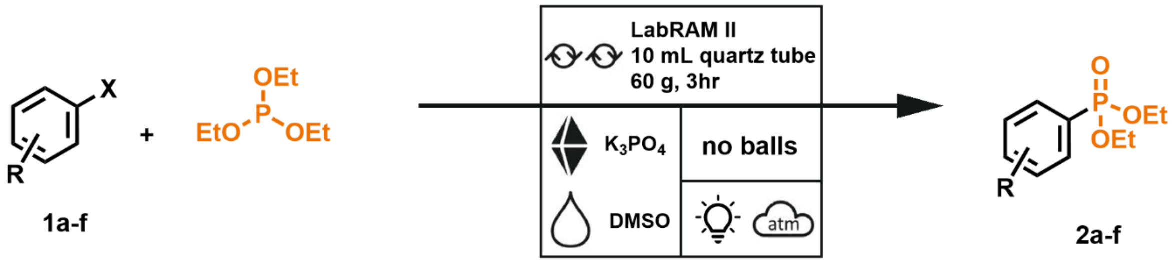

In a typical reaction, 0.38 mmol of the aryl halide, 3 eq. triethyl phosphite, 1.15 g potassium phosphate and dimethyl sulfoxide as liquid-assisted grinding agent (LAG, η = 1) were filled into a quartz glass tube (l = 6.6 cm; d = 2 cm) enclosed by two PFA lids. The reaction mixture was mixed at 60 g for 3 h in the LabRAM II under irradiation of twelve LEDs with λ = 272 nm in a custom designed photoreactor PR1. After the reaction, the crude reaction mixture was taken up in 30 mL ethyl acetate and sonicated for 2 min, before being washed with 30 ml of brine. The aqueous phase was extracted two more times with 30 ml ethyl acetate. The combined organic layers were dried over $MgSO_4$ and concentrated under reduced pressure. A slightly yellow, oily liquid was received of which the yield was determined by ^{1}H-NMR spectroscopy using an equimolar amount of dibromo methane (26 µL) as internal standard.

Upscaling: For a threefold upscaling experiment, we prepared three identical reaction mixtures containing 0.38 mmol 4-iodotoluene, 3 eq. triethyl phosphite, 1.15 g potassium phosphate and dimethyl sulfoxide as liquid-assisted grinding agent (LAG, η = 1) filled into a quartz glass tube (l = 6.6 cm; d = 2 cm) enclosed by two PFA lids. The three reaction mixtures were mixed simultaneously at 60 g for 3 h in the LabRAM II under irradiation of twelve LEDs with λ = 272 nm in a custom designed photoreactor PR1. Therefore, the vessels were placed in the outer holder positions in front of one LED array each. After the reaction, the crude reaction mixtures were combined and taken up in 50 mL ethyl acetate and sonicated for 2 min, before being washed with 50 ml of brine. The aqueous phase was extracted two more times with 50 ml ethyl acetate. The combined organic layers were dried over $MgSO_4$ and concentrated under reduced pressure. A slightly yellow, oily liquid was received of which the yield was determined by ^{1}H-NMR spectroscopy using an equimolar amount of dibromo methane (78 µL) as internal standard.

3.2. General procedure for photochemical CDHC reaction in the RAM (G2)

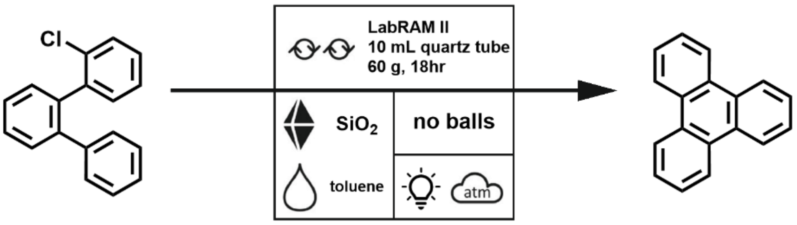

In a typical reaction, 0.56 mmol of 2-(o-chloro) terphenyl (150 mg, 1 eq.), 0.9 g silica as bulk material and toluene as liquid-assisted grinding agent (LAG, η = 1) were filled into a quartz glass tube (l = 6.6 cm; d = 2 cm) enclosed by two PFA lids. The reaction mixture was mixed at 60 g for 18 h in the LabRAM II under irradiation of twelve LEDs with λ = 272 nm in a custom designed photoreactor PR1. After the reaction, the crude reaction mixture was taken up in 30 mL DCM and sonicated for 2 min, before the suspension was separated by centrifugation. The liquid phase was decanted into an extraction funnel and was washed with 30 ml of brine. The remaining solid was

washed with 30 ml DCM and again separated by centrifugation, repeating the whole process two more times. The combined organic layers were dried over $MgSO_4$ and concentrated under reduced pressure. Slightly yellow needles were received of which the yield was determined by ^{1}H-NMR spectroscopy using an equimolar amount of dibromo methane as internal standard.

4. Characterization

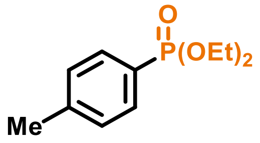

Compound 2a

Compound 2a was synthesized following the general procedure **G1** yielding 97% from 4-iodotoluene and 89% from 4-bromotoluene according to ^{1}H-NMR spectroscopy using dibromo methane as an internal standard. The reaction mixture obtained from the iodo-bearing substrate was purified by flash chromatography using toluene/ethyl acetate (100:0→ 20:80) as an eluent yielding in 60% of 2a (51 mg, 0.23 mmol).

^{1}H NMR (400 MHz, $CDCl_3$) δ 7.74 – 7.64 (m, 2H), 7.30 – 7.22 (m, 2H), 4.19 – 3.97 (m, 4H), 2.39 (s, 3H), 1.30 (t, J = 7.1 Hz, 6H). ^{13}C NMR (101 MHz, $CDCl_3$) δ 143.03, 131.96 (d, J = 10.2 Hz), 129.33 (d, J = 15.3 Hz), 125.18 (d, J = 190.0 Hz), 62.08 (d, J = 5.1 Hz), 21.77, 16.45 (d, J = 6.5 Hz).^{31}P NMR (162 MHz, $CDCl_3$) δ 19.73. NMR data matches closely to the ones reported in literature.[1]

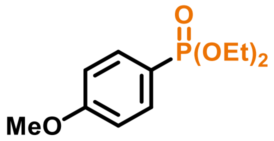

Compound 2b

Compound 2b was synthesized following the general procedure **G1** yielding 86% from 4-iodoanisol according to ^{1}H-NMR spectroscopy using dibromo methane as an internal standard.

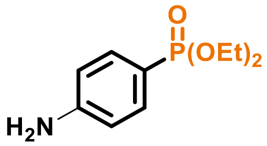

Compound 2c

Compound 2c was synthesized following the general procedure **G1** yielding 46% from 4-iodoanillin according to ^{1}H-NMR spectroscopy using dibromo methane as an internal standard.

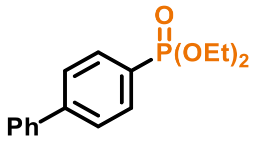

Compound 2d

Compound 2d was synthesized following the general procedure **G1** yielding 46% from 4-iodobiphenyl according to ^{1}H-NMR spectroscopy using dibromo methane as an internal standard.

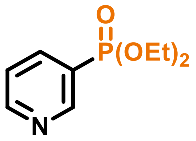

Compound 2e

Compound 2e was synthesized following the general procedure **G1** yielding 38% from 3-bromopyridin according to ^{1}H-NMR spectroscopy using dibromo methane as an internal standard.

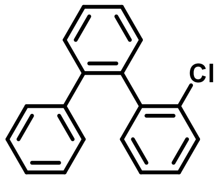

Compound 3

Compound 3 was synthesized and purified according to literature.[2] The purity was assured by ^{1}H-NMR spectroscopy.

^{1}H NMR (400 MHz, $CDCl_3$) δ 7.48 – 7.45 (m, 2H), 7.42 (ddd, *J* = 7.5, 5.1, 3.6 Hz, 1H), 7.38 – 7.32 (m, 2H), 7.32 – 7.27 (m, 1H), 7.20 – 7.13 (m, 6H), 7.12 – 7.07 (m, 2H). The NMR data closely matches the ones reported in literature.[2]

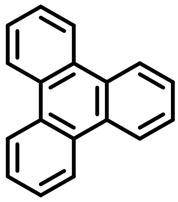

Compound 4

Compound 4 was synthesized following the general procedure **G2** yielding 77% according to ^{1}H-NMR spectroscopy using an equimolar amount of dibromo methane (40 µL) as an internal standard. The product was further purified by recrystallization (0.25 ml/mg) in ethanol giving 74% pure triphenylene (95 mg, 0.42 mmol).

^{1}H NMR (400 MHz, $CDCl_3$) δ 8.75 – 8.61 (m, 4H), 7.80 – 7.55 (m, 4H). ^{13}C NMR (101 MHz, $CDCl_3$) δ 129.94, 127.36, 123.45. The NMR data matches closely to the ones reported in literature.[2]

5. Nuclear Magnetic resonance spectra

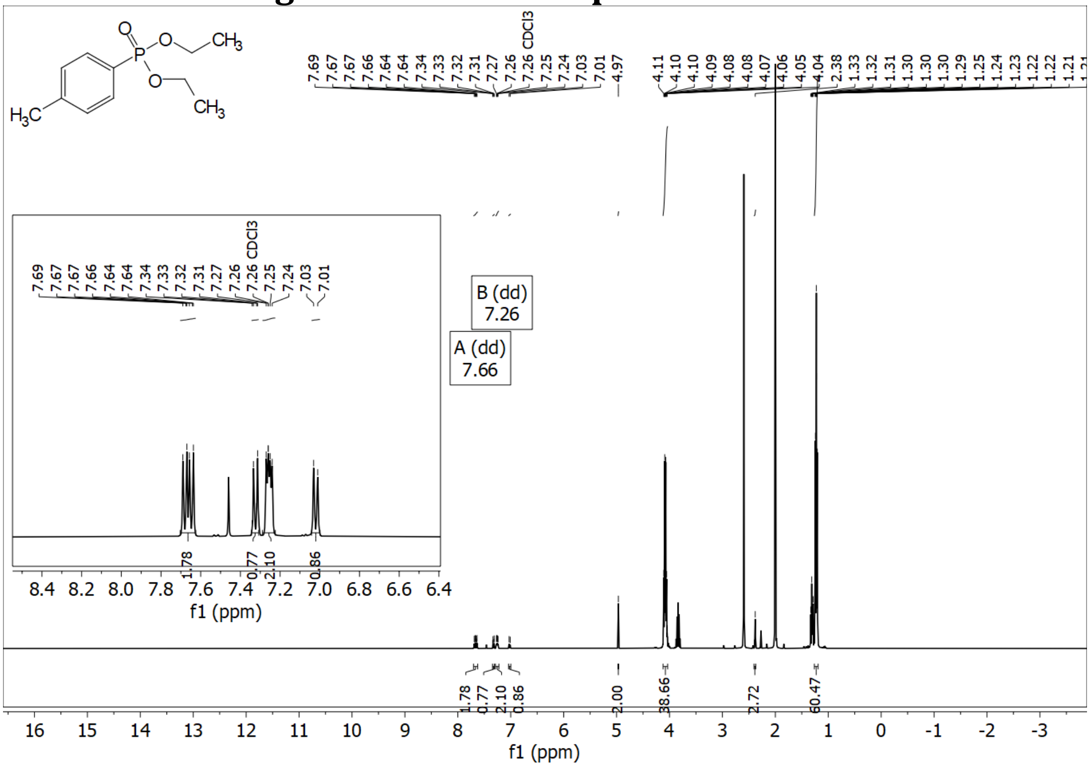

Figure S 7: ^{1}H-NMR spectrum of 4-(diethoxyphosphoryl) toluene (2a) obtained from 4-iodotoluene under optimized conditions before purification in $CDCl_3$ measured at 400 MHz. An equimolar amount of dibromo methane (4.97 ppm) was added to the crude reaction mixture to calculate the yield.

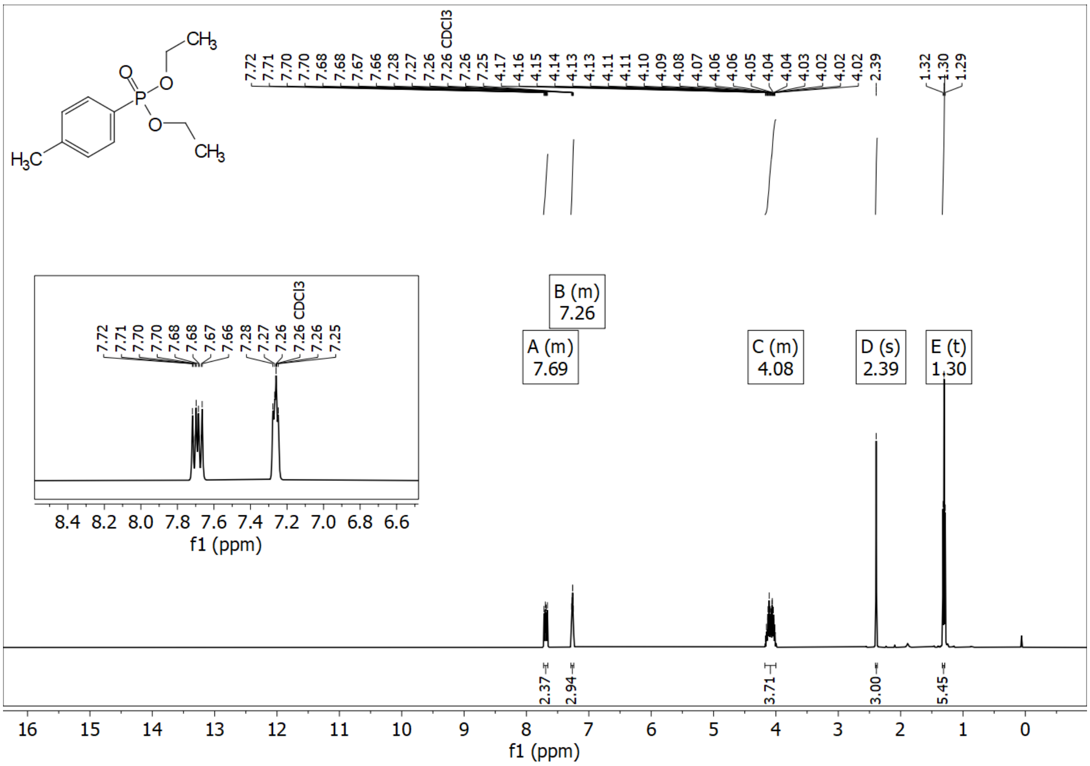

Figure S 8: ^{1}H-NMR spectrum of 4-(diethoxyphosphoryl) toluene (2a) obtained from 4-iodotoluene after purification in $CDCl_3$ measured at 400 MHz. Signal at 7.27 ppm is overlapping with $CDCl_3$ (7.26 ppm) causing an error in the integration.

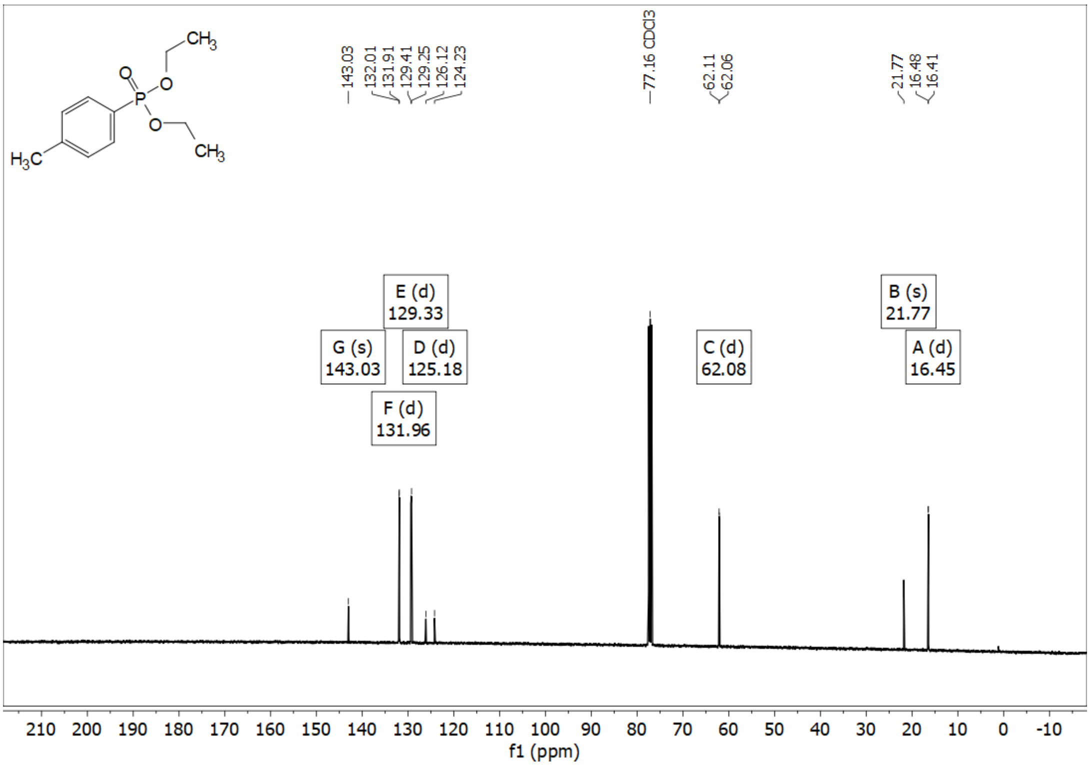

Figure S 9: ^{13}C-NMR spectrum of 4-(diethoxyphosphoryl) toluene (2a) obtained from 4-iodotoluene after purification in $CDCl_3$ measured at 101 MHz.

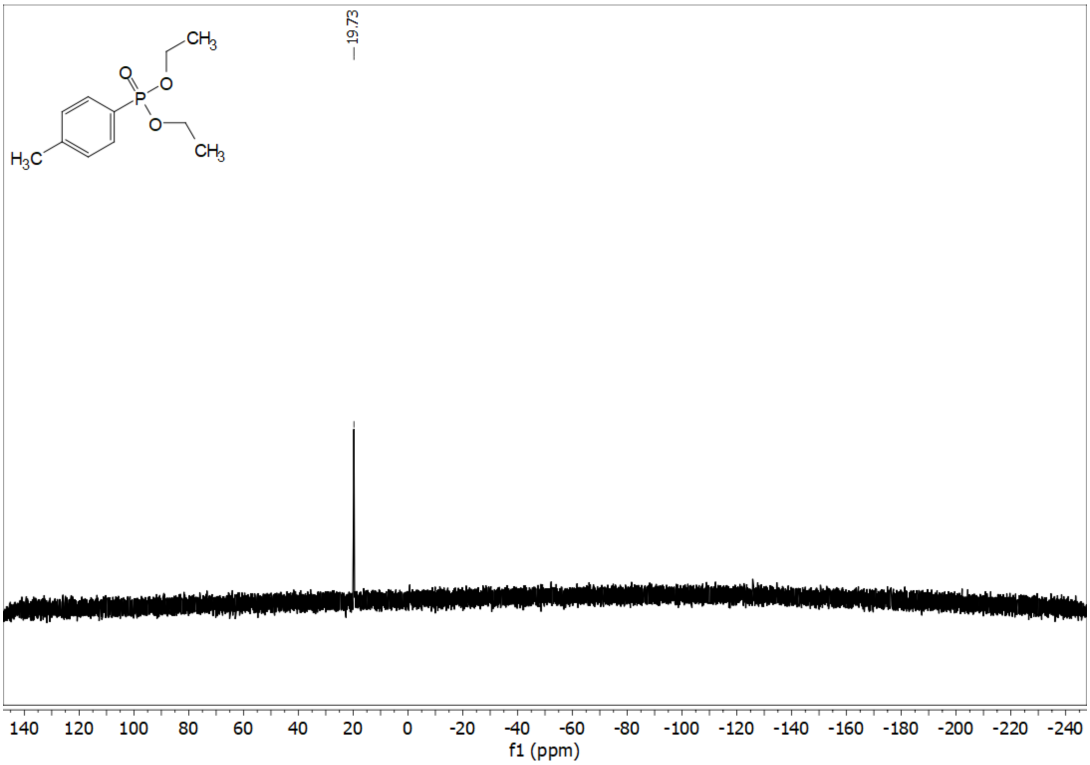

Figure S 10: ^{31}P-NMR spectrum of 4-(diethoxyphosphoryl) toluene (2a) in $CDCl_3$ measured at 162 MHz.

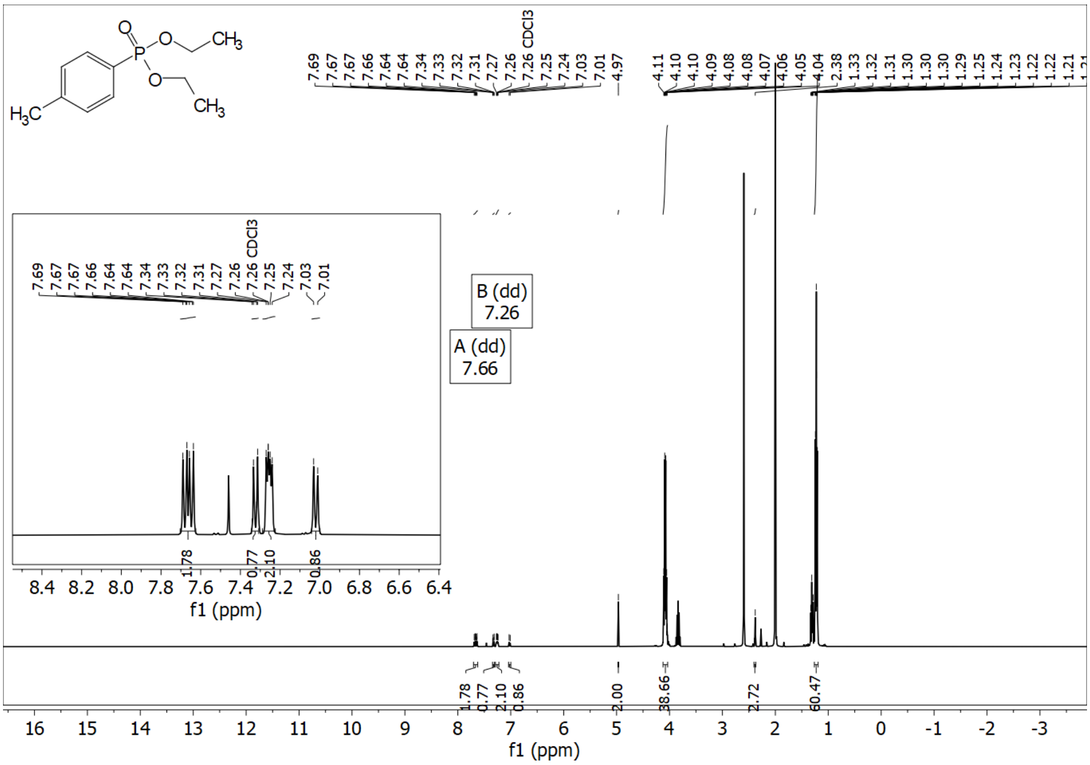

Figure S 11: ^{1}H-NMR spectrum of 4-(diethoxyphosphoryl) toluene (2a) obtained from 4-bromotoluene before purification in $CDCl_3$ measured at 400 MHz. An equimolar amount of dibromo methane (4.97 ppm) was added to the crude reaction mixture to calculate the yield.

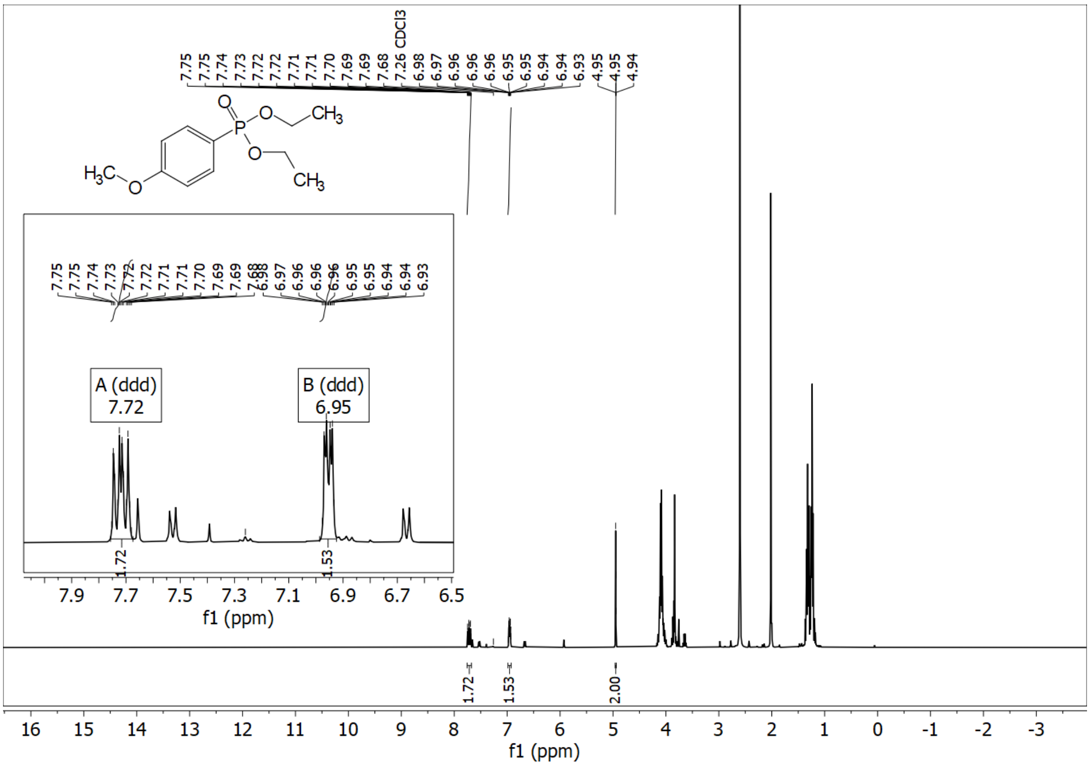

Figure S 12: ^{1}H-NMR spectrum of 4-(diethoxyphosphoryl) anisole (2b) obtained from 4-iodoanisole under optimized conditions before purification in $CDCl_3$ measured at 400 MHz. An equimolar amount of dibromo methane (4.95 ppm) was added to the crude reaction mixture to calculate the yield.

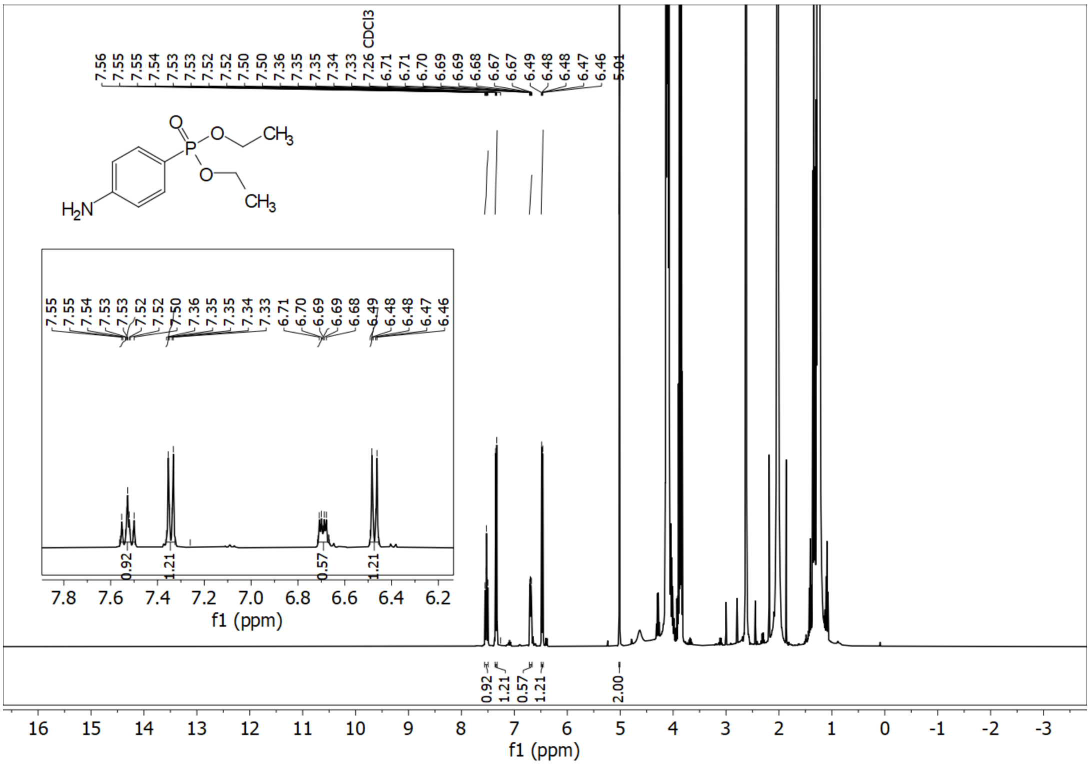

Figure S 13: ^{1}H-NMR spectrum of 4-(diethoxyphosphoryl) anilline (2c) obtained from 4-iodoanilline under optimized conditions before purification in $CDCl_3$ measured at 400 MHz. An equimolar amount of dibromo methane (5.01 ppm) was added to the crude reaction mixture to calculate the yield.

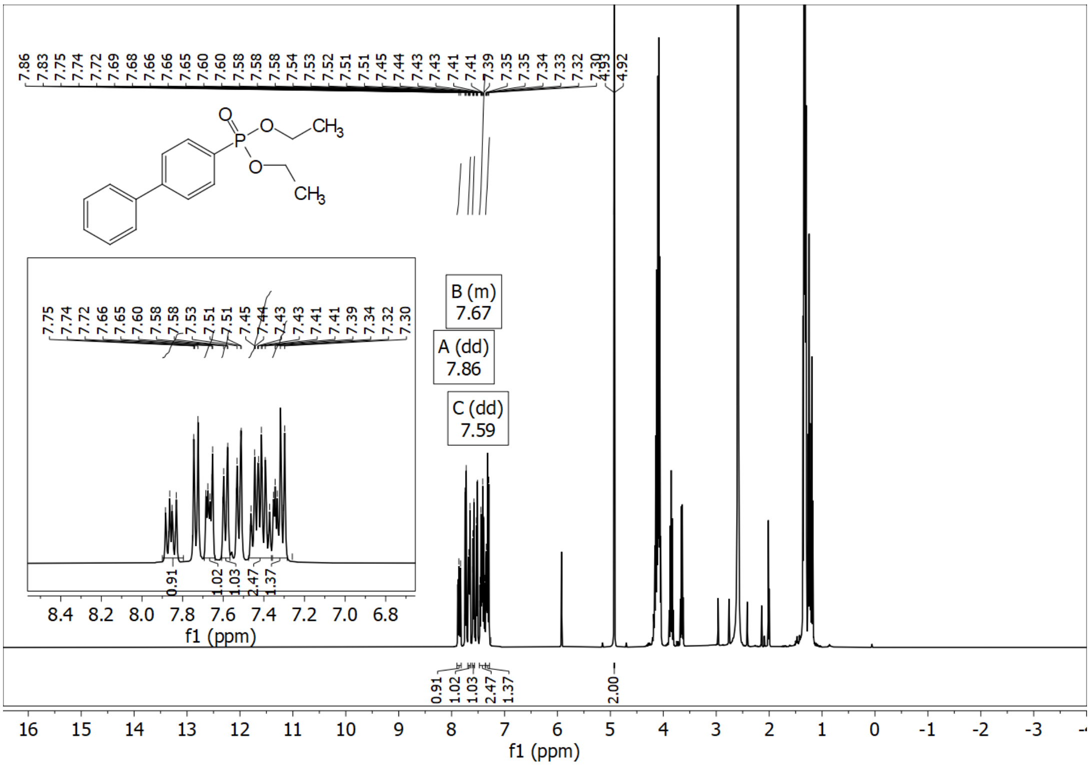

Figure S 14: ^{1}H-NMR spectrum of 4-(diethoxyphosphoryl)-1,1'-biphenyle (2d) obtained from 4-iodo-1,1'-biphenyl under optimized conditions before purification in $CDCl_3$ measured at 400 MHz. An equimolar amount of dibromo methane (4.93 ppm) was added to the crude reaction mixture to calculate the yield.

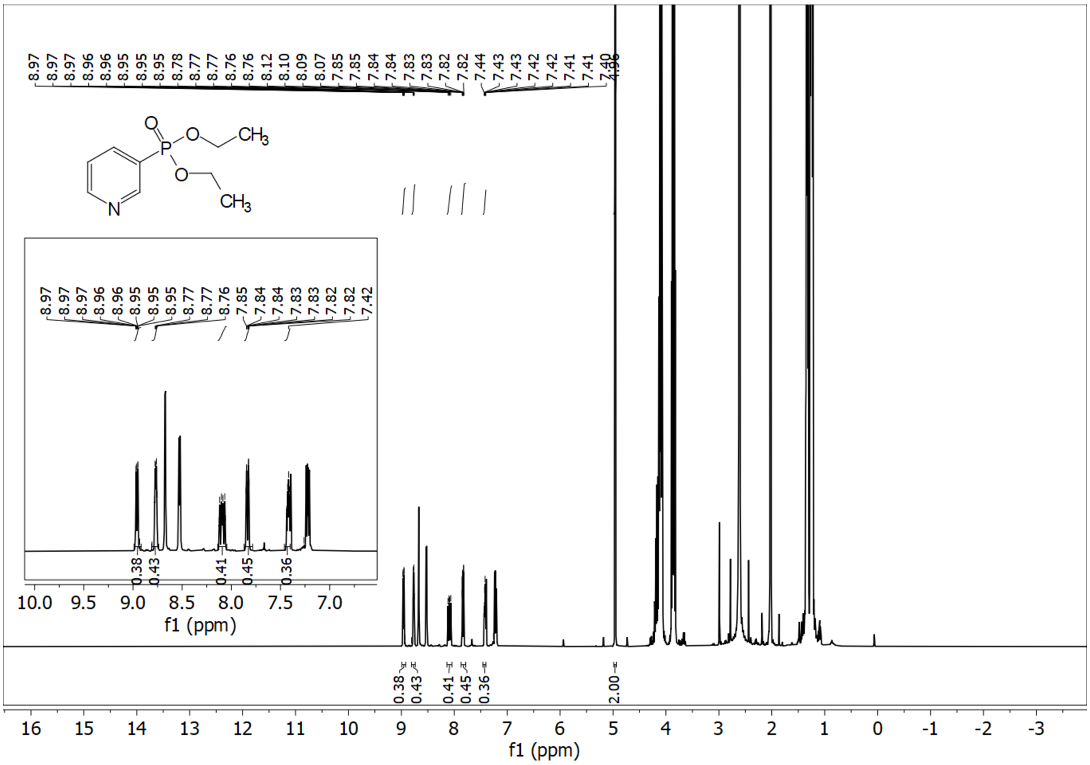

Figure S 15: ^{1}H-NMR spectrum of 3-(diethoxyphosphoryl) pyridine (2a) obtained from 3-bromopyridine under optimized conditions before purification in $CDCl_3$ measured at 400 MHz. An equimolar amount of dibromo methane (4.96 ppm) was added to the crude reaction mixture to calculate the yield.

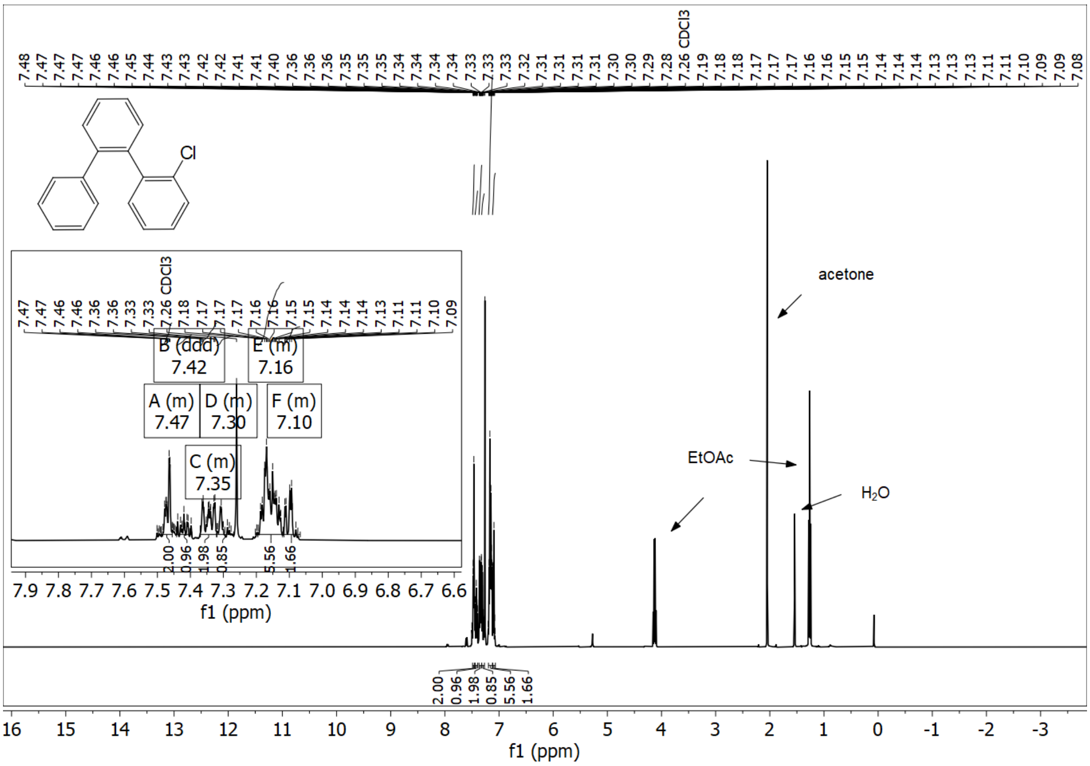

Figure S 16: ^{1}H-NMR spectrum of o-(2-chloro) terphenyl in $CDCl_3$ measured at 400 MHz. Remaining solvent signals are marked.

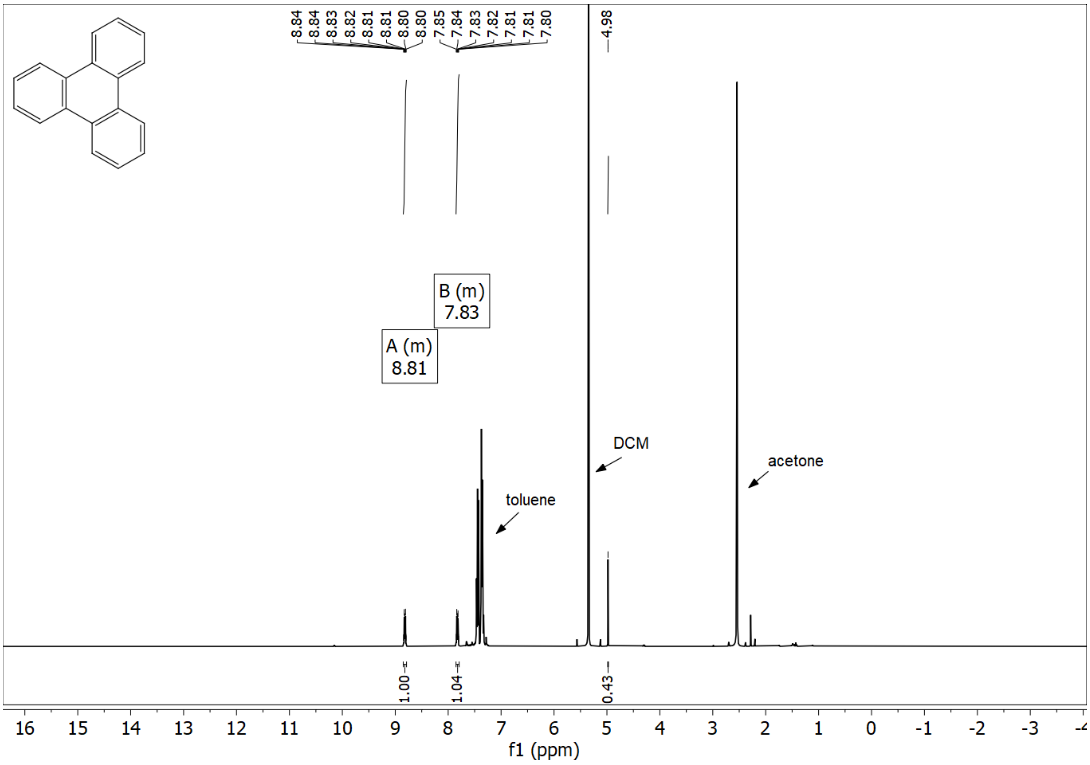

Figure S 17: ^{1}H-NMR spectrum of triphenylene before purification in $CDCl_3$ measured at 400 MHz. An equimolar amount of dibromo methane (4.98 ppm) as an internal standard is added to calculate the yield. Remaining solvent signals are marked.

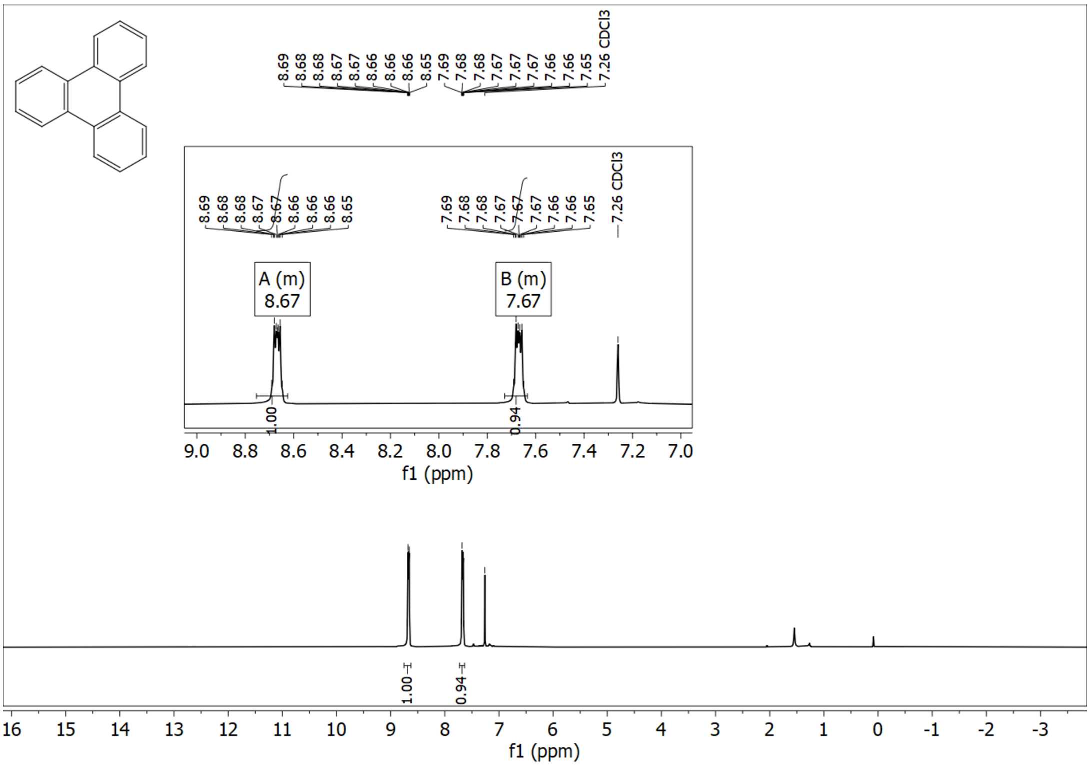

Figure S 18: ^{1}H-NMR spectrum of triphenylene after purification in $CDCl_3$ measured at 400 MHz.

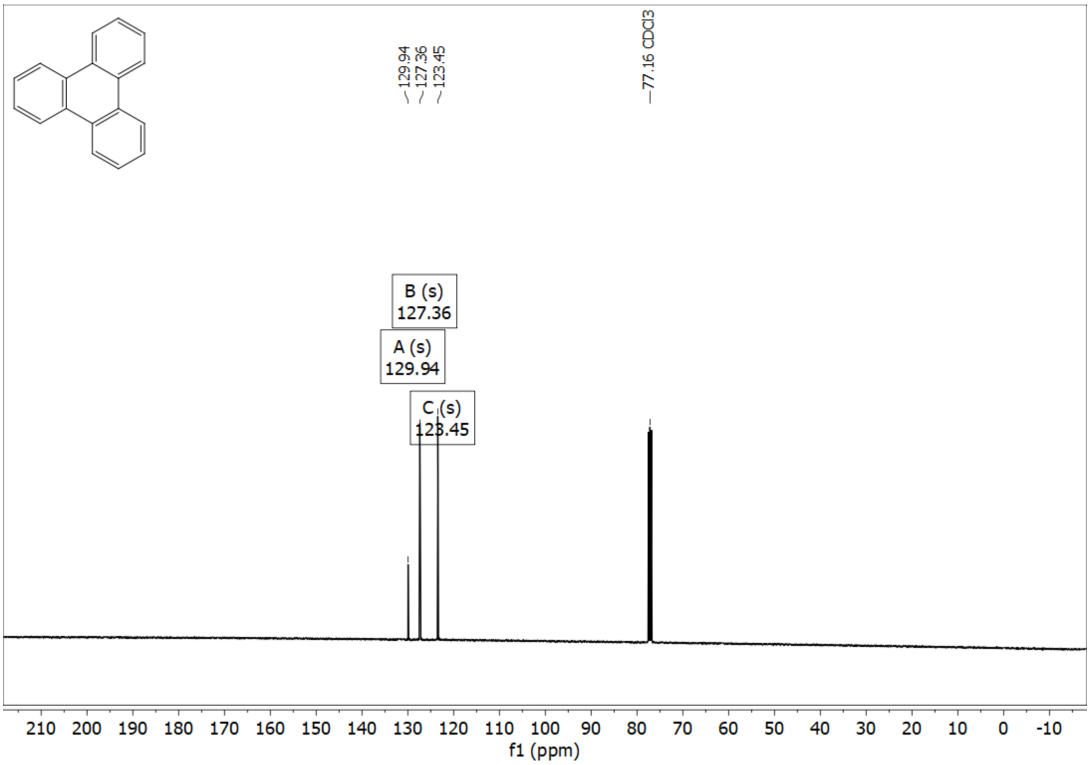

Figure S 19: ^{13}C-NMR spectrum of triphenylene after purification in $CDCl_3$ measured at 101 MHz.

6. References

[1] N. G. W. Cowper, C. P. Chernowsky, O. P. Williams, Z. K. Wickens, *J. Am. Chem. Soc.* **2020**, *142*, 2093.

[2] D. M. Baier, C. Spula, S. Fanenstich, S. Grätz, L. Borchardt, *Angew. Chem. Int. Ed.* **2023**, *62*, e202218719.
